# Bulk serum extracellular vesicles from stressed mice show a distinct proteome and induce behavioral and molecular changes in naive mice

**DOI:** 10.1371/journal.pone.0308976

**Published:** 2024-08-15

**Authors:** Melisa C. Monteleone, Silvia C. Billi, Lorena Abarzúa-Catalán, Roberto Henzi, Eliana M. Fernández, Thilo Kaehne, Ursula Wyneken, Marcela A. Brocco

**Affiliations:** 1 Instituto de Investigaciones Biotecnológicas, Universidad Nacional de San Martín (UNSAM)–Consejo Nacional de Investigaciones Científicas y Técnicas (CONICET), San Martín, Argentina; 2 Escuela de Bio y Nanotecnologías (EByN), Universidad Nacional de San Martín, San Martín, Argentina; 3 Facultad de Medicina, Centro de Investigación e Innovación Biomédica CiiB, Universidad de los Andes, Santiago, Chile; 4 IMPACT, Center of Interventional Medicine for Precision and Advanced Cellular Therapy, Santiago, Chile; 5 Institute of Experimental Internal Medicine, Medical School, Otto von Guericke University Magdeburg, Magdeburg, Germany; Free University of Berlin, GERMANY

## Abstract

Chronic stress can trigger several pathologies including mood disorders for which no clear diagnostic molecular markers have been established yet. Attractive biomarker sources are extracellular vesicles (EVs). Evs are released by cells in health and disease and contain genetic material, proteins and lipids characteristic of the cell state. Here we show that Evs recovered from the blood of animals exposed to a repeated interrupted stress protocol (RIS) have a different protein profile compared to those obtained from control animals. Proteomic analysis indicated that proteins differentially present in bulk serum Evs from stressed animals were implicated in metabolic and inflammatory pathways and several of them were previously related to psychiatric disorders. Interestingly, these serum Evs carry brain-enriched proteins including the stress-responsive neuronal protein M6a. Then, we used an in-utero electroporation strategy to selectively overexpress M6a-GFP in brain neurons and found that M6a-GFP could also be detected in bulk serum Evs suggesting a neuronal origin. Finally, to determine if these Evs could have functional consequences, we administered Evs from control and RIS animals intranasally to naïve mice. Animals receiving stress EVs showed changes in behavior and brain M6a levels similar to those observed in physically stressed animals. Such changes could therefore be attributed, or at least in part, to EV protein transfer. Altogether these findings show that EVs may participate in stress signaling and propose proteins carried by EVs as a valuable source of biomarkers for stress-induced diseases.

## Introduction

Prolonged exposure to stressful situations is known to be related to the development of many diseases including mood disorders [1]. Particularly, major depressive disorder (MDD) has become the leading cause of disability worldwide with females being more than twice as likely to develop MDD as males [2]. Solid evidences support the idea that chronic stress precipitates depressive states in humans and induces depressive-like behaviors in animal models by altering neuroendocrine, physiologic, morphologic and behavioral parameters and levels of several mRNAs and proteins [3–5]. Many of these proteins have been studied as potential markers for MDD and others mood disorders. For example, serum levels of brain derived neurotrophic factor (BDNF) are reduced in patients in comparison to healthy controls and are responsive to antidepressant treatments [6,7]. Despite all this knowledge, clinicians do not have powerful reliable diagnostic tools (biomarkers) to identify people at risk due to exposure to chronic stress or patients suffering from a mood disorder so far.

One of the proteins affected by stress is the transmembrane glycoprotein M6a [3,4,8], abundantly expressed in the central nervous system (CNS) [9]. M6a promotes neurite outgrowth, filopodium/spine and synaptic density formation [10–12]. Interestingly, chronically stressed mice displayed a reduction in transcript hippocampal levels for M6a, an effect that can be reversed by treatment with the antidepressant drug tianeptine[10]. Notably, M6a is detected in extracellular vesicles (EVs) isolated from human saliva and from rodent (mice and rats) serum [4,13]. However, the cellular origin of the M6a-loaded EVs remains unknown. EVs are secreted by almost all cell types and their molecular cargo, consisting of proteins, RNA species, and lipids, changes with physiological and pathological conditions. EVs can permeate biological barriers, such as the blood-brain barrier (BBB) [14,15]. Thus, serum EVs can proceed from any tissue/organ/cell, including the brain. These features have drawn attention to the EV molecular content as disease biomarker reservoir particularly from inaccessible areas such as the brain.

We and others have demonstrated that the molecular cargo of EVs retains bioactivity once taken up by recipient cells, affecting their function [4,16,17]. Also, there are several reports depicting EV-based treatments. However, results are controversial. While some authors suggest beneficial effects of disease-derived EVs [18,19], others show the opposite, that disease-derived EVs are capable of reproducing the features of the disease in the recipient organism [20,21].

Taking this into consideration, we wonder whether the response to chronic stress involves the release of extracellular vesicles with differential content into the periphery (i.e., blood). To this, we characterized the protein content of bulk serum EVs isolated from mice exposed to chronic stress and found potential biomarkers. We have also demonstrated the presence of brain proteins, including M6a, in circulating EVs. Finally, we assessed whether these vesicles administered to naïve individuals could impact their behavior.

## Materials and methods

### Animals

**Fig 1:**
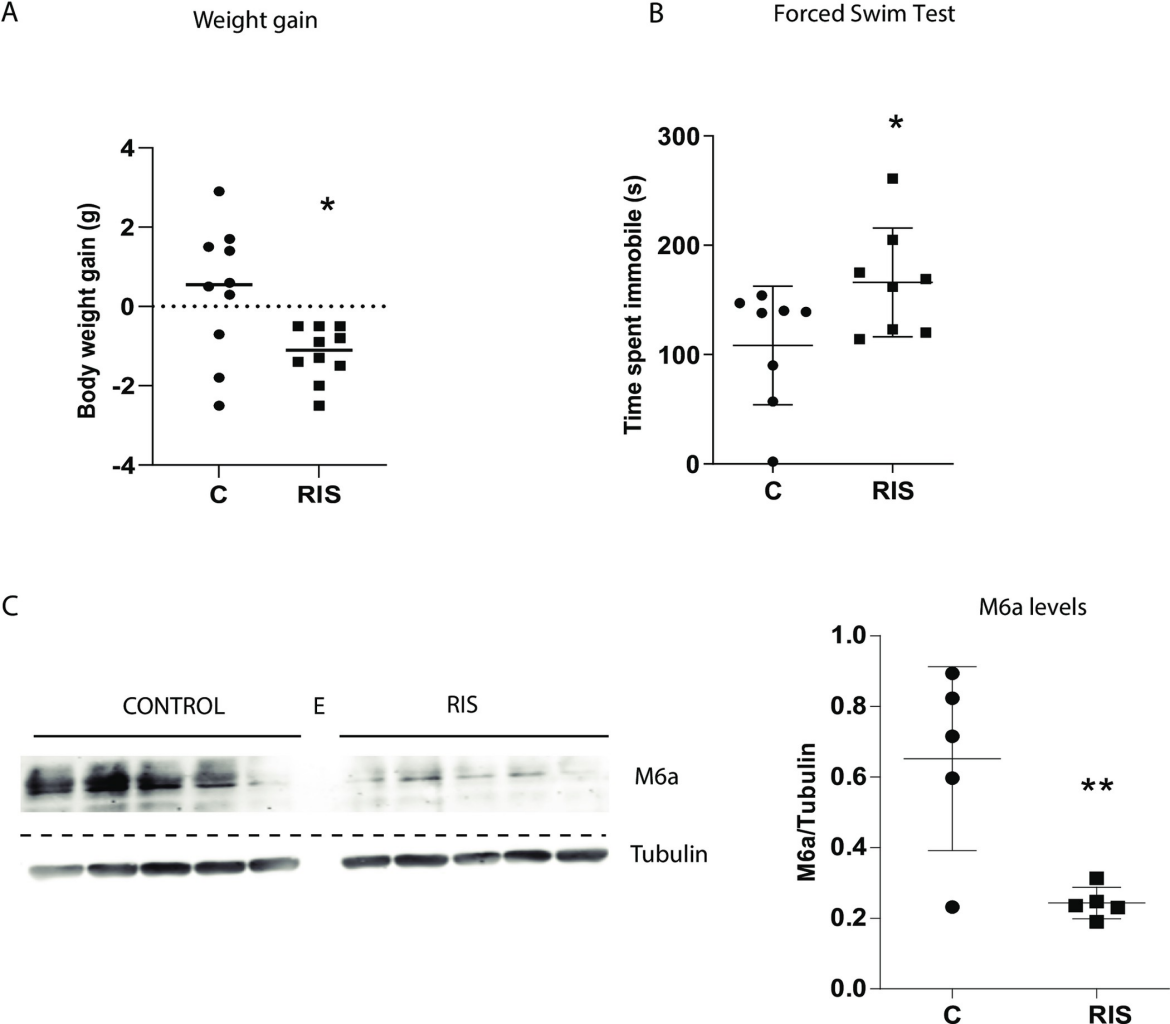
The Repeated Interrupted Stress (RIS) protocol induces changes in body weight, behavior and hippocampal M6a levels. One day after the end of RIS, stressed (RIS, n = 10) and non-stressed (C, n = 10) mice were tested. **A.** RIS induced a significant reduction in body weight gain, *p<0.01. **B.** RIS significantly increased the time spent immobile in the forced swim test (FST), *p<0.05. **C.** RIS also caused a significant reduction in the expression levels of the hippocampal glycoprotein M6a (p<0.01). Each lane represents one individual. Equal amounts of proteins were loaded per lane. Dashed line indicates where the image was cropped. An empty naïvee (E) was left between control and RIS animals. Full scans can be seen in S1 Fig. Prior to antibody hybridization membranes were cut at 40kDa using the Molecular Weight marker as reference. Membranes were treated with antibodies anti-M6a and with anti- tubulin for normalization purposes. Data are presented as mean ± SD, for statistical analysis a two-tailed t-Student test was used.

**Fig 2:**
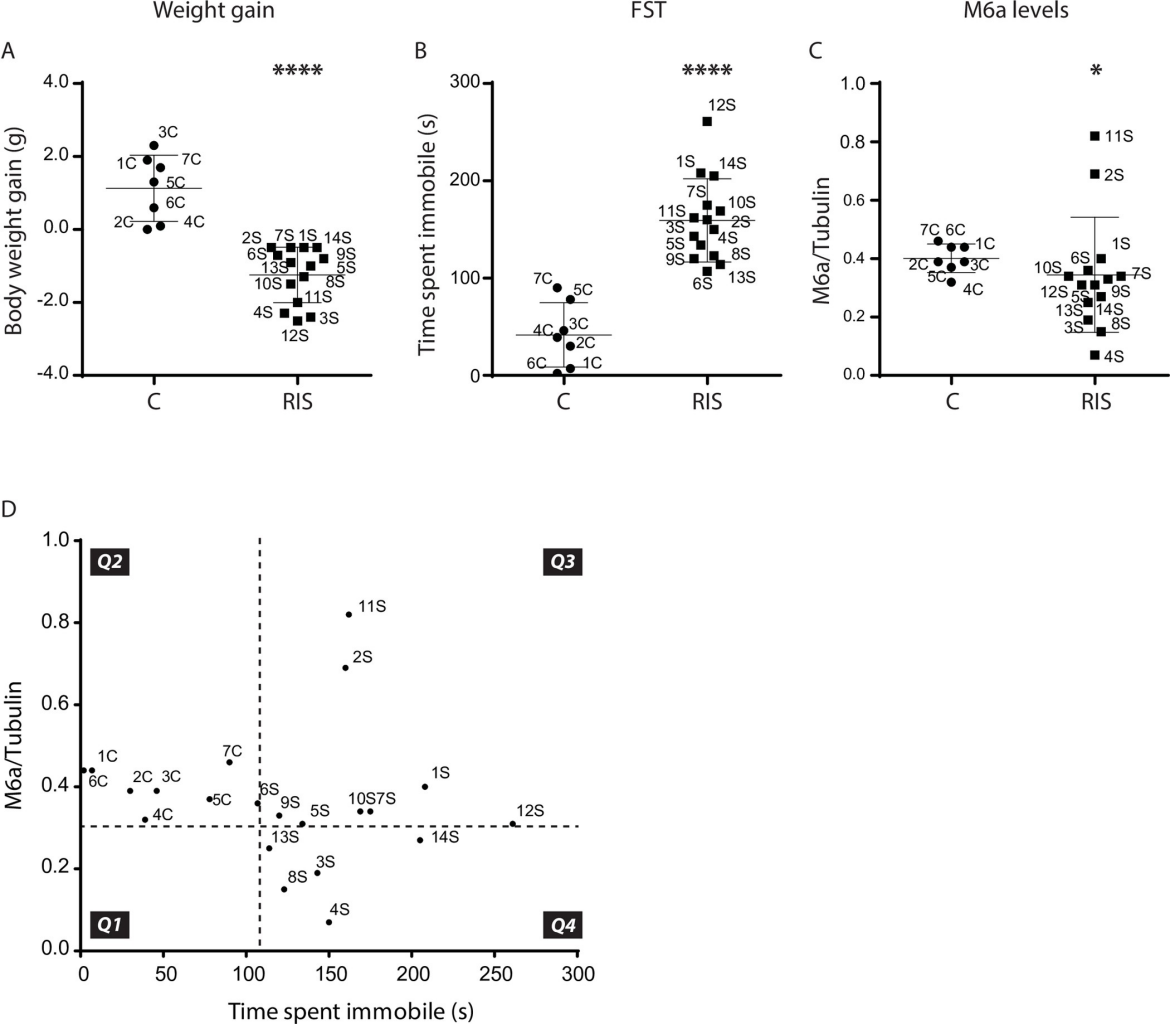
RIS leads to divergent individual stress responses. Body weight gain, FST and M6a levels were evaluated in a new cohort of animals. **A.** RIS induced a general reduction in body weight gain (****: p<0.0001). **B.** RIS increased the time spent immobile in the FST (****: p<0.0001). **C.** RIS also reduced M6a hippocampal levels (*: p<0.05). However, a great interindividual variability was observed. **D.** The M6a/Tubulin level vs. time spent immobile was plotted to classify animals. Dotted lines indicate cut-off values and were calculated as the mean value plus two standard deviations (time spent immobile) or minus (M6a/Tubulin) two standard deviations. All control animals that exhibited low time spent immobile and high M6a levels hence are located in the Q2 quadrant. Animals in the Q4 quadrant displayed low levels of M6a and high time spent immobile, and were considered stressed. Animals that were subjected to RIS but displayed high levels of M6a (Q3) were classified as less sensitive to stress.

C57Bl/6J female mice between 2.5 and 3 months (approximately 22–25 g) of age were used for the stress protocols. For in-utero electroporation, pregnant Sprague–Dawley rats were used. The progeny grew until young adulthood, and their blood was obtained. The experimental procedures were supervised and approved by the Committee on the Ethics of Animal Experiments of the Universidad Nacional de San Martin (CICUAE-UNSAM #003/2021) for mice and by the Bioethical Committee of the Universidad de los Andes(for rats). The National Institute of Health’s Guide for the Care and Use of Laboratory Animals was followed. Animals were maintained with *ad libitum* access to food and water under a 12-h-light/-dark cycle at 22°C ± 1°C. All experiments were designed to reduce the number of animals in each experiment. For the chronic stress experiments n = 6-10/group was used. Sample size was calculated using the resource equation method in which a value “E”, which should lie between 10 and 20, is measured as follows: E = Total number of animals − Total number of groups [22]. For experiment from Fig 1: E = 18; for experiment from Fig 2: E = 19, for experiment in Fig 5: E = 12. Two independent experiments were used to assess chronic stress effects and for proteomic analysis. For the instillation experiment a new set of animals was used (n = 5/group). These animals received EVs from a third independent chronic stress protocol carried out exclusively for EV isolation. In this case, 5 animals/group were included.

This work is reported in accordance with ARRIVE guidelines.

### Experimental design

Naive mice were stressed chronically for three weeks. The Interrupted Stress Protocol (RIS) protocol was based on the one described by Zhang et al. [23] as it helps avoid mice habituation to uninterrupted stress. Weight was daily recorded. Mice were individually placed into transparent plastic tubes fitted closely to their body for 4 h per day, five days per week followed by a two-day break with no stress. Age-matched unstressed animals were used as controls. At the end of the stress protocol, the behavioral tests (see below) were carried out. Twenty-four hours later, blood was collected from the orbital sinus. After that, animals were sacrificed and brains were removed for hippocampus isolation. Hippocampal tissue was homogenized in a lysis buffer (150mM NaCl, 20 mMTris-HCl [pH 7.5], 1% Triton X-100, 1mM EDTA). Blood samples(700 μl) were incubated at 37°C for 30 min to allow clot formation, then they were centrifuged for 5 min at 12000 xg (EppendorfMiniSpin Plus) to obtain serum samples.

### Forced Swim Test (FST)

Animals were placed into 4l-plastic recipients (13 cm diameter, 24 cm height) filled with 22°C water, 17 cm deep. Sessions were video taped. The time spent immobile during a 6 min testing period was measured. Immobility, evaluated by a blind observer, was defined according to the criteria described previously [24].

### Protein quantification, Western blots and antibodies

Protein content was quantified using the Bradford dye-binding method (Bio-Rad Laboratories). 30ug of total protein was loaded in each lane. Primary antibodies used: polyclonal rabbit anti- body against C-terminus of M6a (1/1000) developed in our laboratory, anti-Flotillin-1 (#610820 BD Transduction Laboratories, 1/1000); anti-Calnexin (#C4731 Sigma Aldrich, 1/2000), anti-CD63 and anti-CD9,1/500 (H5C6-S and B2C11-S respectively Developmental Studies Hybridoma Bank (DSHB), Iowa City, United States),anti-NeuN (#6712A, EMD Millipore, Burlington, MA, United States, 1/2000), anti-GFAP (#Z0334, Dako, Santa Clara, CA, United States, 1:1000) and anti-GFP (#ab1218, Abcam, Cambridge, MA, United States, 1:750) Membranes were scanned using the Odyssey system or by enhanced chemiluminescence immunodetection method (ECL-WB). When possible, the membrane was cut after transference taking as reference the molecular weight marker. Full images of scanned membranes as well as where and how the membranes were cut are shown in S4–S6 Figs.

### EV isolation

EVs were isolated from serum samples (~150–200 μl of serum per animal). All Evs were isolated by ultracentrifugation. For proteomics, where we analyzed each sample individually, serum was centrifuged at 2,000 xg for 20 minutes, then at 20,000 xg for 30 min and finally at 22 psig (approximately 120,000 xg) in the air-driven ultracentrifuge system (Airfuge, Beckman). All centrifugations were done at 4ºC. Tubes (Beckman Coulter, Cat number 344718) were used. EV pellets were resuspended in 120 μl sterile PBS and stored at -20°C until further use.

For the isolation of Evs from the offspring of electroporated dams, 6 ml of serum was obtained per rat and treated as mentioned above and then ultracentrifuged at 120,000 xg (Beckman Ti70 rotor) for 120 min. Temperature was set at 4°C. Polycarbonate tubes (Beckman Coulter, Cat number 355654) were used. EV pellets were resuspended in 50 μl sterile PBS, then quantified and stored at -20°C until further use. Evs accepted markers Flotillin-1, CD9 and CD63 were assessed as well as the negative marker calnexin (S3A Fig). Other contaminant quantities such as APO A/B and albumin were estimated from LC-MS/MS data. The decision to use Western blot analysis over LC-MS/MS or vice versa was primarily influenced by feasibility considerations. Serum Evs isolated by these methods display typical EV characteristics: size range between 30–100 nm and round cup shaped morphology under TEM analysis, as we have shown previously [4,25].

### EV intranasal administration

Serum Evs isolated from control (n = 5) or RIS (n = 5) mice were pooled. Each pool was then separated into 5 doses to intranasally administer to 5naïve (not subjected to any experimental manipulation other than EV administration) mice. Evs were prepared from 400 μl serum each (almost all serum from a mouse was used to administer to one paired-aged mouse, as reported earlier [26]). Each mouse received 70 μg of total protein (quantified by Bradford assay). As a control experiment 5 mice received PBS (10 μl) via intranasal. Using a small pipette, Evs suspension was gradually released into the nostrils in five doses on alternating sides of the nose as 2-μl drops spaced 2 minutes apart (10 μl was administered in each nostril). Careful observation was done to assure that all solution volume was entering the nose and nothing was swallowed or sneezed. In addition, to assess EV biodistribution, a small group of animals received by the same administration method Evs stained with the lipophilic dye DiIC_18_(7); 1,1′-dioctadecyl-3,3,3′,3′-tetramethylindotricarbocyanine iodide(DiR)and24h scanned (Odyssey Scanning System) (S1 Fig). Full body scans show that while animals that received PBS, boiled Evs or DiR alone displayed most of the fluorescence in the body, whilst animals that received intact Evs (from either treatment, control or RIS) showed fluorescence in the tail, suggesting that Evs reached circulation (S1A Fig). When we analyzed the brains, mice that received boiled Evs or DiR alone retained the fluorescence in the brain, while animals that received intact Evs displayed less brain fluorescence, suggesting that Evs were able to move along different tissues (S1B Fig). The qualitative analysis of DiR signal in serum samples from instilled animals shows a detectable DiR signal (arrows in S1C Fig) only in the serum of mice instilled with Evs (intact or not) because DiR stains lipidic membranes (S1C Fig).

Twenty-four hours after EV instillation, animals were weighed and evaluated in the FST and then left undisturbed for a week, weighed again and evaluated in the FST. Finally, mice were euthanized to collect hippocampi.

### In-utero electroporation

Electroporation was performed at embryonic stage E18.5 to 19.5 [27]. To anesthetize pregnant rats, xylazine (5 mg/kg) and ketamine (50 mg/kg) were administered via intraperitoneal (i.p.) injection. Uterine horns were exposed and plasmid mixtures (2.7 μg of each plasmid mixed with 0.5 μL of Fast Green [1 mg/mL, Sigma-Aldrich]) were injected into the left lateral ventricle using pulled glass capillaries (P97, Sutter Instruments) connected to a pressure Pico pump (PV830, World Precision Instruments). The plasmids were pPBTalpha1-PBase (approximately 2 μg/μl), which is driven by the neuron-specific Talpha1 alpha-tubulin (Talpha1) promoter combined with: (1) pBCAG-M6a-GFP (approximately 2 μg/μl), that is, driven by the strong ubiquitous promoter cytomegalovirus early enhancer/ chicken beta-actin. The pPBTalpha1-PBase and pBCAG-GFP plasmids were kindly donated by Joseph LoTurco and a stable transgene expression in neuron progenitors was achieved through the piggyBac transposon system [28]. For electroporation, a 60- to 70-V electric pulse was delivered across a pair of oval electrodes (1 × 0.5 cm) with the positive pole placed on the lateral surface of the left cerebral hemisphere. Finally, the electroporated fetuses were born and grew to collect brain samples for immunofluorescence and blood collection.

### Immunofluorescence

Electroporated rats of 250 g were anesthetized with ketamine (50 mg/kg) and xylazine (5 mg/kg) and were perfused intracardially with 0.9% saline followed by 4% paraformaldehyde. Then the brains were removed, cryopreserved, and cut in 30-μm frozen coronal sections using a Microm HM 525 cryostat (Thermo Fisher Scientific). Serial sections from electroporated and non- electroporated rats were incubated with blocking solution (phosphate buffered saline [PBS] pH = 7.4, 0.25% w/v Triton X-100, 5% w/v horse serum [#16050130, Gibco-Invitrogen, San Diego, CA], 5% w/v bovine serum albumin [BSA]) for 1 hour at room temperature. Then they were incubated overnight at 4°C with the corresponding primary antibodies (anti-NeuN and anti-GFP) diluted to 1:1000 with PBS pH 7.4, 0.25% w/v Triton X-100, 1% w/v horse serum, and 1% w/v BSA. Then the sections were incubated for 1 hour at room temperature with diluted (1:1000) secondary antibody coupled to fluorescent probes (Alexa anti goat 488 or Alexa anti mouse 555). Omission of the primary antibody during incubation was used as control. Slides were cover slipped by using Vectashield mounting medium (Dako, Agilent) and inspected under an epifluorescence microscope (Nikon, ECLIPSE TE2000U) or a confocal microscope (Leica SP8) to study co-localization by using the multidimensional acquisition software.

### Proteomic analysis

#### Sample selection

EV samples from mice belonging to stress and control group were selected according hippocampal M6a levels and FST immobility time. Samples from the control group belonged to those mice showing M6a levels and immobility times higher and lower than higher than cut-off values, respectively (cut-off values were settled as the mean plus two standard deviations, dotted lines on Fig 2D). Samples from control individuals that did not meet these criteria were excluded. Stressed animals were classified as stressed (low M6a levels and high FTS immobility time) or less susceptible to stress (Q3 group, high M6a levels and high FTS immobility time). Thus, EV samples from stressed animals were analyzed separately.

Isolated EVs (20 ug protein per sample) were suspended in Laemmli’s buffer and sent for proteomic analysis.

#### Normalization

EV preparations were subjected to sodium dodecyl sulfate–polyacrylamide gel electrophoresis (SDS-PAGE) to fine-adjust protein amounts for label free proteome analysis. After staining the gel with colloidal Coomassie Blue according to manufacturer’s protocol the optical density of each sample lane was determined with a calibrated gel scanner in transmission mode and the relative protein amount was calculated.

#### Digestion and fractionation

Each lane was divided into 3 sections to perform in-gel digestion in an adapted manner according to Shevchenko, 1996 [29]. Collected peptides were dried in a vacuum centrifuge.

#### Mass spectrometry

LC-MS/MS was performed on a hybrid dual pressure linear ion trap/orbitrap mass spectrometer (LTQ OrbitrapVelos Pro, Thermo Scientific, San Jose, CA, USA) equipped with an Ultimate 3000-nLC Ultra HPLC (Thermo Scientific, San Jose, CA, USA). Dried peptide fractions were dissolved in 10μl0.1% TFA and subjected to a 75 μm I.D., 25 cm PepMap C18-column, packed with 2 μm resin (Dionex, Germany). Separation was achieved by applying a gradient from 2% ACN to 35% ACN in 0.1% formic acid (FA) over a 130 min gradient at a flow rate of 300 nl/min. The LTQ OrbitrapVelos Pro MS exclusively used CID-fragmentation when acquiring MS/MS spectra, consisting of an orbitrap full MS scan followed by up to 20 LTQ MS/MS experiments (TOP20) on the most abundant ions detected in the full MS scan. The essential MS settings were as follows: full MS (FTMS; resolution 60,000; m/z range 400–2000); MS/MS (Linear Trap; minimum signal threshold 500; isolation width 2 Da; dynamic exclusion time setting 30 s; singly charged ions were excluded from selection). Normalized collision energy was set to 35%, and the activation time was set to 10 ms.

#### Data processing

Raw data processing and protein identification of the high resolution orbitrap datasets were performed with *de novo* sequencing algorithms of PEAKS Studio 8.0 (Bioinformatics Solutions Inc., Waterloo, Canada) using the SwissProt database. The false discovery rate was set to <1% with the appropriate threshold. PEAKS includes an unique addition algorithm to detect post translational modified peptides. LFQ analysis was performed using PROGENESIS QI for Proteomics (Nonlinear Dynamics/Waters Corporation, Milford, MA, USA).

The STRING database [30] was used to generate protein-protein interaction networks. The enrichment of Gene Ontology (GO) terms (Gene Ontology Consortium, 2015) within proteomic datasets was also performed with STRING. SynGO database was consulted to find human synapse-related proteins (https://www.syngoportal.org/about.html.

S1 Table shows the bibliography reporting the link between most candidates with nervous system diseases.

### Statistical analysis

Statistical analysis and graphs were carried out with GraphPad Prism Version 5.00.288. All data were subjected to normality and equal variance testing using IS version 2010 (Infostat software, GrupoInfoStat, FCA, Universidad Nacional de Córdoba, Argentina). Results were reported as mean ± SD. When two groups were compared the t-test or its non-parametric version Mann-Whitney U-test was used. ANOVA or its non-parametric version was used for more than two group comparisons. For all tests, a P < 0.05 was considered statistically significant.

Images were processed with the ImageJ or FiJi software.

## Results

### Repeated interrupted stress (RIS) changes protein profile of bulk serum EVs

To study if stress can modify the protein content of EVs, we first characterized the effects of chronic stresson female mice. Female mice were chosen as there is a higher incidence rate of global prevalence of depressive disorders in women than men (World Health Organization data). To induce stress, we used a three-week RIS protocol in which animals are subjected to stress daily with a two-day interruption. This short interruption is used as a tool to stall or reverse habituation [23]. As we have previously reported [4], RIS induced a significant reduction in mouse body weight gain (Fig 1A p = 0.02) which is a pathophysiological indicator of stress generally accepted in the literature [31,32]. To evaluate stress effects on behavior, we tested the learned helplessness in the forced swim test (FST), which measures passive coping strategy to acute inescapable stress. We found a significant increase in the time spent immobile in RIS mice compared with control ones (Fig 1B p = 0.04). Regardless of the interpretation of the meaning of the behavior in the FST, i.e., measurement of behavioral despair or stress-coping strategies, our findings indicate that the FST was sensitive to RIS. Beyond the global results observed, it is worth noting the great interindividual variability in both tests, which has already been observed in other animal models [33].

To characterize RIS mice at a molecular level, we analyzed the expression of the protein M6a. We found a significant decrease in M6a hippocampal levels after RIS (p<0.01 Fig 1C).

Next, we studied if there were differences in the proteins carried in EVs from control (EVs-C) vs. EVs from RIS(EVs-RIS) mice through proteomic analysis. We carried out a second stress assay and the selection of EV samples was based on immobility time in the FST and M6a levels, stress sensitive measurements well documented using this stress protocol (Fig 1 and [4,23,34]). Body weight gain was also considered. Fig 2A and 2C shows the distribution of control and stressed individuals regarding the three parameters evaluated. As to body weight gain, all control individuals displayed a positive body weight change where as all stressed individuals exhibited a negative body weight change (Fig 2A). Regarding immobility time in the FST and M6a levels, most stressed mice showed significantly higher (FST) and/or lower (M6a levels) values than control mice. However, some of the stressed individuals (e.g., 6S and 13S for FST and 1S and 6S for M6a levels) showed values similar (or even higher in the case of M6a levels such as individuals 2S and 11S) to those of the control individuals (Fig 2B and 2C). This reinforces the idea of interindividual variability in the stress response already depicted [35]. To select samples taking into account this variability, we plotted the levels of M6a vs. immobility time in the FST and we separated the individuals into 4 groups (quadrants) using as cut-off values the mean plus two standard deviations (dotted lines in Fig 2D). This plot shows that all control animals showed high M6a levels and low immobility time (Q2). On the opposite, in the Q4 quadrant, stressed animals that displayed high immobility time and low M6a hippocampal levels were located (Fig 2D, 4th quadrant Q4). Stressed individuals that showed only one of the parameters different from control, i.e., higher levels of M6a (quadrant Q3) could be considered less susceptible to stress. Since differences in coping strategies could be due to different expression patterns and differences in genetic, epigenetic and neurochemical factors[36], we characterized and compared the protein profile of serum EVs from the three groups: control (Q2), stress (Q4) and less susceptible to stress (Q3), separately.

**Fig 3:**
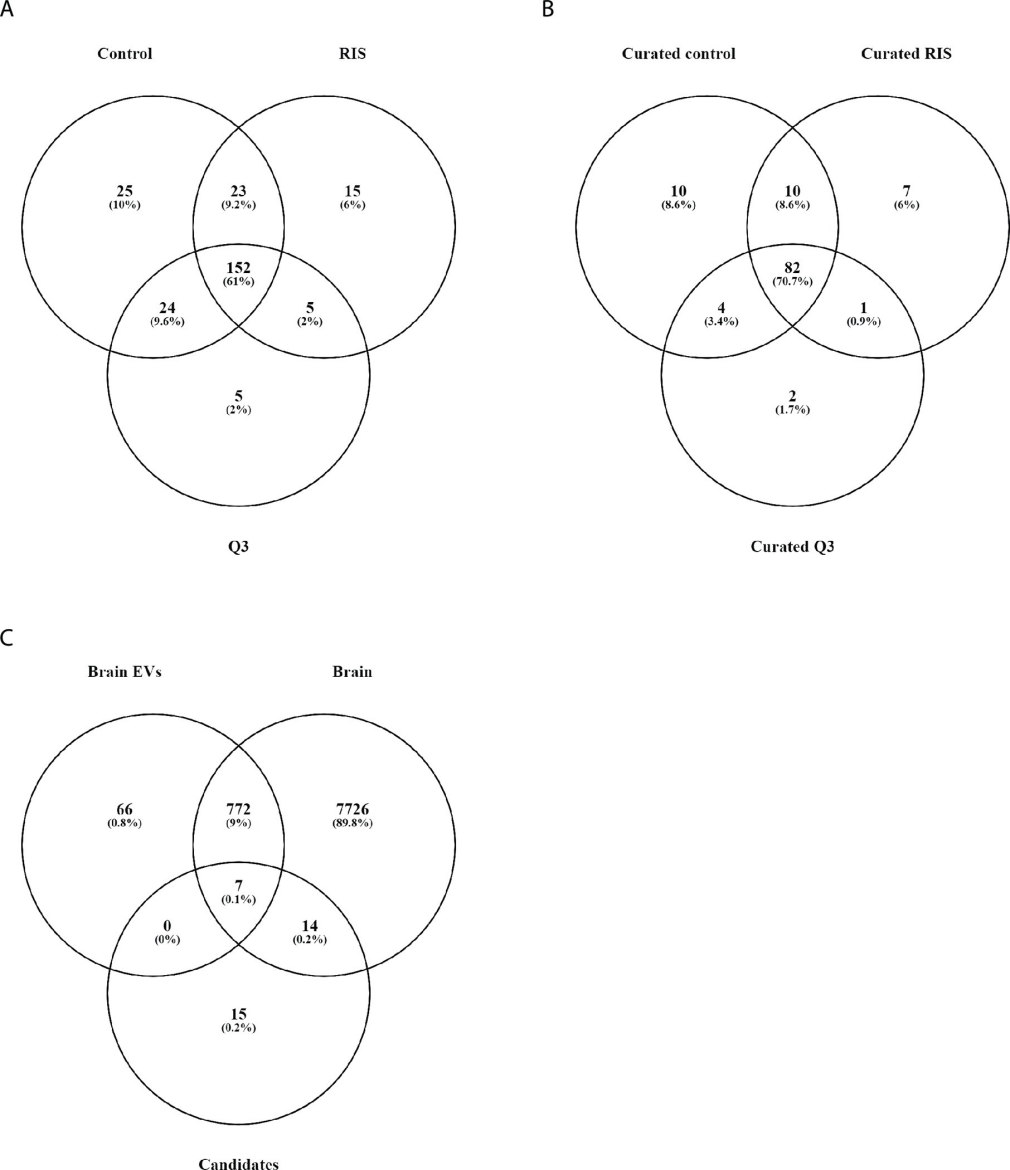
Qualitative analysis Venn diagram showing proteins detected in EVs-C, EVs-RIS or EVs-Q3. Before **A.** and after **B.** exclusion of keratins and immunoglobulin chains. Here, the following 10 proteins were exclusively detected in EVs-C: DESP: Desmoplak in, F13B: Coagulation factor XIII B chain, H2A2A/2B/2C: histone 2A type A, B and C, PLAK: Junction plakoglobin, 1433Z: 14-3-3 zeta/delta protein, FBLN3: EGF-containing fibulin-like extracellular matrix protein 1, UBB: Ubiquitin B, G3P Glyceraldehyde-3-phosphate dehydrogenase. Seven proteins were exclusively detected in EVs-RIS: ACTBL: Beta-actin-like protein 2, FA5 and FA10: coagulation factors V and X, PSA6: Proteasome subunit alpha type-6, B3AT: Band 3 anion transport protein also known as Solute carrier family 4 (anion exchanger) member 1 (SLC4A1), APOC3: Apolipoprotein C-III, RET4: Retinol-binding protein 4 and two proteins were exclusively detected in EVs-Q3: CES1D: Carboxylesterase 1D and PSA7 proteasome subunit alpha type-7.**C.** Proteomic analysis of extracellular vesicles (EVs) shows that many (21/36) of the candidate proteins are expressed in the brain. Venn analysis of the differentially expressed proteins between the experimental conditions (Candidates, identified after quantitative proteomic approach (LFQ-analysis) detailed in supplementary file sheet 8), the proteome of mouse brain extracellular vesicles (Brain EVs) and mouse brain proteins (Brain, http://www.mousebrainproteome.com/).

First, in a qualitative approach, we identified the most abundant proteins detected in each group. Setting the threshold of the identification score (-10lgp) to 100 (p = 1E-10) and after manual curation(i.e. manual revision and correction or deletion)of the list to exclude duplicated proteins, keratins and immunoglobulin chains, almost 100 proteins were left for each group (S1 File sheets 1–6, Fig 3A before and 3B after curation). Since the majority (82 proteins) was common among groups, no significant differences were found in molecular function, biological processes or cellular component. Qualitative analysis also showed that some proteins could be detected only in one experimental condition: 10 in EVs derived from control animals (Evs-C), 7 in Evs-RIS and 2 in less susceptible to stress Q3-Evs (S1 File sheet 7 and Fig 3B).

Moving forward to a label free quantitative proteomic approach (LFQ-analysis) to find different protein levels in the EVs from the different groups, we subjected the acquired LC-MS/MS data to an analysis using the PROGENESIS QI software (Nonlinear Dynamics/Waters). We obtained 50 proteins with differential expression among groups and therefore potential candidates (S1 File sheet 8). Among these proteins20 were higher in EVs-C than in EVs-RIS and 2 were lower; 8 were higher in EVs-C than in EVs- less susceptible to stress (Q3) and 5, lower. Also, differences between RIS and less susceptible to stress (Q3) were found: 10 proteins were higher in EVs-RIS than in EVs-less susceptible to stress (Q3) and 5, lower. Since there were repeated proteins between groups, we manually deleted them and obtained a list of 36 unique proteins differentially expressed between treatments that could be potential candidates (S1 Table and S1 File sheet 9).

Beyond heterogeneity of the molecules found, interestingly, 21 out of 36 are expressed in the brain: COL4A2, COL6A2, CSF1R, DDI2, FAT2, GGA1, ITIH2, IRS2, JPH1, PDZD8, SLC25A20, SNAP23, TENM2, USP19, **CLU, DPP6, HBA, HBB-B1, NOMO1, PRDX2 and SYN2** with the last seven proteins (highlighted in bold) were also detected in mouse brain EVs [37] (Fig 3C). In addition, CLU, SYN2 and SNAP23 are included in the SynGO Database [38], a resource providing synaptic gene annotations (S1 File sheet 9). This reinforces the idea that brain information can be transferred to the periphery e.g., forming part of bulk serum EVs.

Using the String database, a protein-protein interaction (PPI) network with the 21 brain protein candidates revealed more interactions than expected by chance (p < 0.05). Although no enriched GO terms were found, several proteins were included in the Focal Adhesion -PI3K-Akt-mTOR-signaling pathway (S1 File sheet 10).

Regarding M6a, thecandidate and stress-sensitive protein, it was evidenced by only one unique peptide (EEQELHDIHSTRSKER) in control and in less susceptible to stress (Q3)-EVs, therefore it was not sufficient to perform any LFQ analysis (at least three unique peptides/protein are required for reliable protein abundance calculation).

### M6a-GFP selectively expressed in neurons can be detected in serum

**Fig 4:**
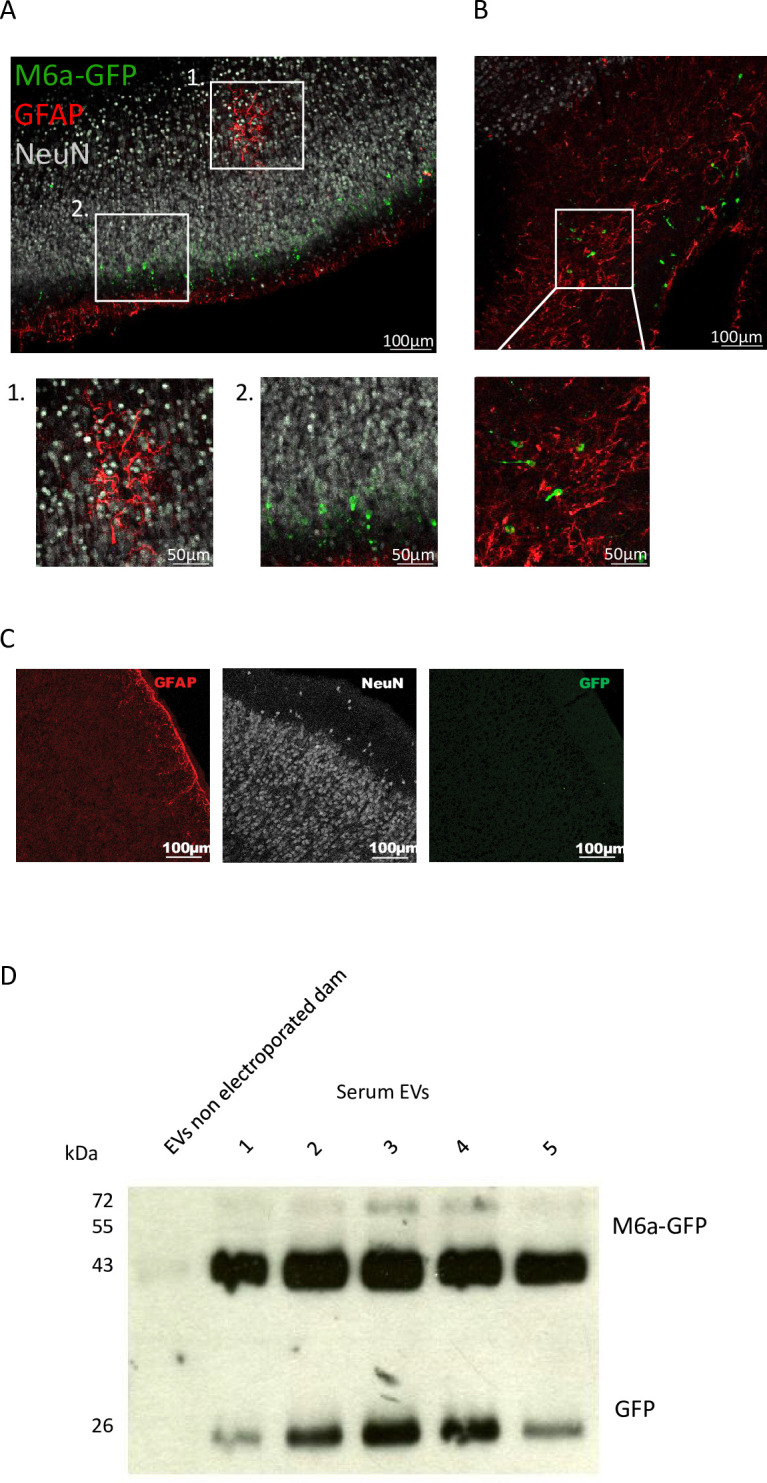
M6a selectively expressed in neurons can be detected in serum. **A**. Representative image of brain (cerebral cortex) slices from postnatal day 5 pups electroporated in-utero Anti-GFAP and anti-NeuN antibodies were used as astrocyte and neuronal markers respectively. M6a tagged with green fluorescent protein (GFP) under the T-alpha promoter was electroporated in-utero to directly target neurons. Immunofluorescence detection of glial fibrillary acid protein (GFAP, red), GFP (green) and the neuronal marker NeuN (gray) in coronal brain slices indicated that cells positive for M6a-GFP signal did not overlap with GFAP positive cells and had neuronal morphology (see magnifications). **B.** Image shown in A rotated to show the molecular cortical layer to dorsal. **C.** Representative image of brain (cerebral cortex) slices from postnatal day 5 non electroporated pups. Immunofluorescence detection of glial fibrillary acid protein (GFAP, red), neuronal marker NeuN (gray) and GFP (green). No GFP signal could be detected **D.** Western blot analysis of EVs isolated from serum of a non-electroporated control rat and 5 adult rats electroporated in-utero with the construct. Anti-GFP antibody was used to detect both GFP (~ 27 kDa) and M6a-GFP (~ 70 kDa). As seen, the recombinant protein was detected in the EVs isolated from the serum of all electroporated animals but not in the control. Full scan can be seen in S5 Fig.

Although M6a has been identified in serum, saliva and CSF [4,13], its putative brain origin has not been studied yet. For this, at embryonic day 18.5, we carried out an in-utero electroporation to transfer plasmid DNA coding M6a-GFP into the lateral ventricle. In this plasmid, the expression of M6a-GFP is under the control of the neuron-specific Tα1 α-tubulin (Tα1) promoter [39].This promoter restricts M6a-GFP expression within neurons [40]. At postnatal day 5, brain slices were immunostained with anti-GFP, anti-NeuN as a mature neuron marker and anti-GFAP as a marker of astrocytes (Fig 4A and 4B). M6a-GFP-positive and GFAP-positive cell were found and did not overlap (Fig 4A inset 1 and Fig 4B) demonstrating the promoter specificity and indicating that glial cells did not express M6a-GFP. NeuN and GFP positive cells colocalized and single GFP-stained cells resembling neurons were observed (Fig 4A and 4B and insets) supporting that brain neurons expressed M6a transferred in-utero. To evaluate if neuronal M6a-GFP could be detected in the periphery, we collected the serum of electroporated rats once they reached adulthood and isolated EVs. The recombinant protein, detected using the GFP antibody (Fig 4C), revealed bands of approximately 55 kDa and 26 kDa corresponding to the M6a-GFP fusion protein and GFP alone respectively. Since M6a-GFP expression is under the control of Tα1, a neuronal-specific promoter and the electroporation was done in the fetal brains restricting protein expression to the brain, these observations suggest that the recombinant M6a detected in serum EVs derives from brain neurons. Thus, we postulate that neuronal status might be assayed using serum.

### EVs from RIS animals induce stress like phenotypes in recipient animals

**Fig 5:**
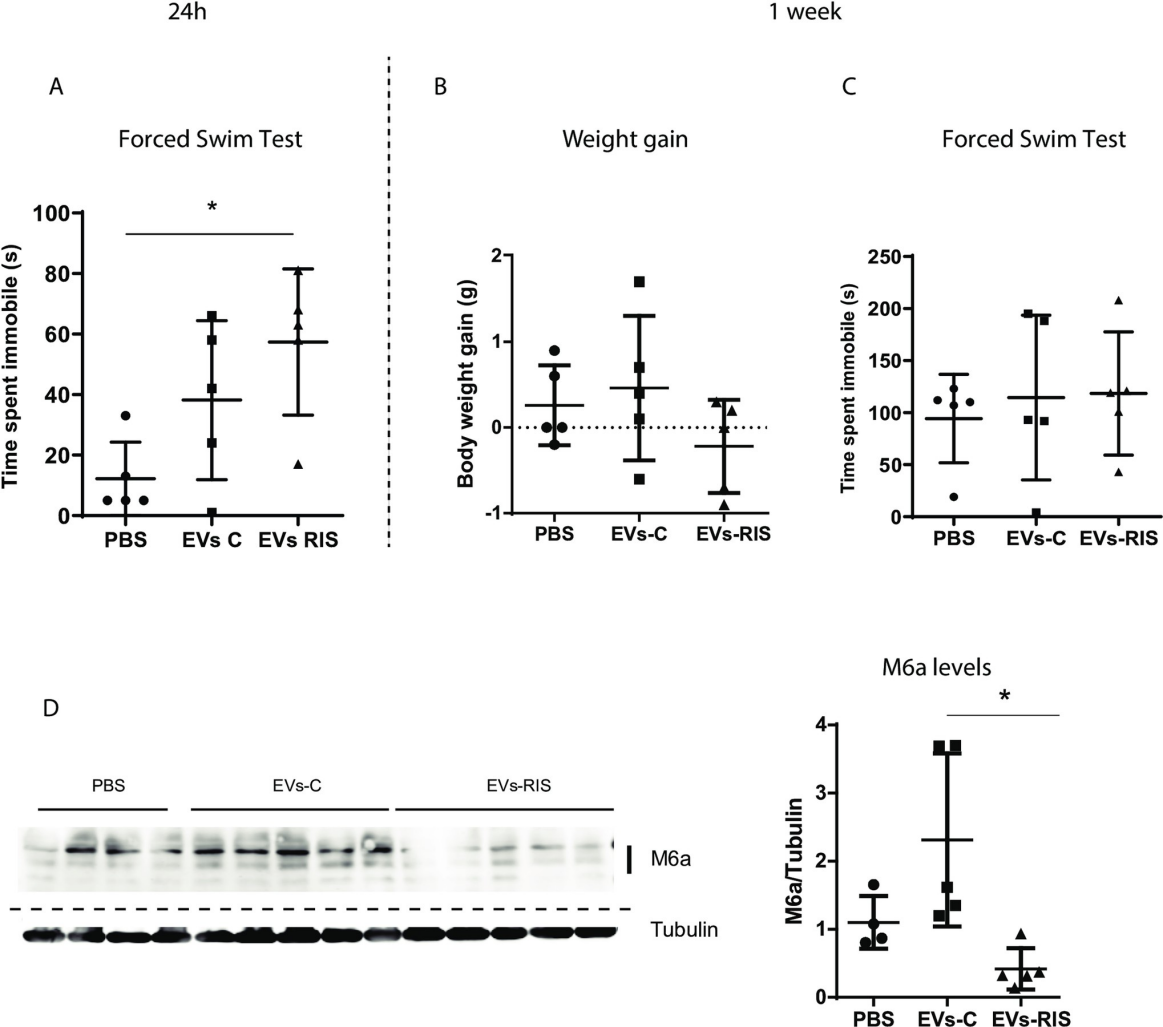
Intranasal administration of bulk EVs isolated from the serum of RIS mice induces behavioral and molecular changes like those observed in stressed animals. Twenty-four hours after EV administration, mice were evaluated with the forced swim test (FST). **A.** Time spent immobile increased in animals that received EVs derived from RIS animals (EVs-RIS) compared with animals that received PBS, *p<0.05.n = 5/group, ANOVA and by Holm-Sidak’s multiple comparisons test. One week after EV administration body weight gain **B**, and **C** FST performance were evaluated. No differences were found among groups. **D.** Western blot and the corresponding densitometric quantification of changes in the content of M6a in hippocampal homogenates one week after EV administration. Each lane represents one individual, n = 5/group. Dashed line indicates where the image was cropped. Prior to antibody hybridization membranes were cut at 40kDa using the Molecular Weight marker as reference. Full scan can be seen in S6 Fig. Equal amounts of proteins were loaded per lane. Tubulin levels were used to normalize protein load. M6a levels were significantly reduced in animals that received EVs-RIS animals compared to those who received EVs-C or PBS (n = 4-5/group, ANOVA followed by Holm-Sidak’s multiple comparisons test *p < 0.05).

Since we found differences in protein content between C- and RIS- EVs, we wonder if this differential content might induce different effects when transferred to naïve individuals. Moreover, we tested the hypothesis that EVs convey enough stress signals to elicit stress-like physiological, molecular and behavioral changes. To this, we transferred EVs from stressed to non-stressed animals. Specifically, serum EVs from RIS (EVs-RIS) and control (EVs-C) mice were administered intranasally only once into naïve, i.e., non-stressed age-paired animals. First, using a small animal subset that received DiR-labelled EVs, we explored EV biodistribution and observed that EVs reached the brain and passed through it as 24 h later, no EVs were found in the brain (See S1A and S1C Fig and Materials and Methods section).We found that 24 h after EV transfer, in comparison with animals that received PBS (vehicle), mice that received EVs-RIS showed an increase in the time spent immobile in the FST (Fig 5A, p = 0.0195). No differences were observed between animals that received EVs from control animals and the PBS group. Animals were then left undisturbed for a week. No differences in body weight gain or time spent immobile in the FST between groups were found (Fig 5B and 5C). However, we did find that animals that received EVs from stressed animals had decreased M6a hippocampal levels compared to animals that received EVs-C (Fig 5D). These results go in line with reports stating that chronic stress reduces M6a hippocampal levels [3,4,41,42].

Noteworthy, while mice receiving EVs-RIS showed similar reduced M6a levels, there was a higher interindividual variability among animals receiving EVs-C and even if we exclude the two individuals with the greatest expression, the differences are still significant (p = 0.0151) between animals that received EVs-C and EVs-RIS. Furthermore, only in mice that received EVs-RIS a slight correlation (p = 0.08) between behavior (FST) and M6a levels was observed (S2 Fig 24h, red dots). Although such correlation was lost a week after EV administration, the animals that received EVs-RIS still displayed the lowest levels of M6a (S2 Fig 1w, red dots).

Altogether these results suggest that Evs carry information that can cause molecular and behavioral changes that recapitulate some of the phenotypes observed in stressed animals.

## Discussion

In this work, the RIS consequences at different levels were demonstrated by physiological (body weight gain), behavioral (FST) and molecular (M6a levels) changes. Although changes were clear, we observed a large interindividual variability (already reported for inbred C57BL/6 mice [43]) that led us to further classify animals. RIS-animals were considered as stress sensitive (Q4) or less sensitive (Q3) according to hippocampal M6a levels: the lower the levels, the more sensitive to stress. Our group has previously demonstrated that chronic stress may modify M6a serum levels in a similar way to what is observed in the hippocampus [4]. Moreover, we have also shown that saliva M6a levels change in depressed women according to their perceived stress scores [13], indicating that M6a is stress-responsive and that it is an interesting candidate as a stress biomarker. In addition, antidepressant treatment in both mouse and human models can modify M6a levels revealed by brain mRNA and by the saliva protein [13,44], suggesting an additional role as a marker for therapy monitor.

It has already been proposed that proteins expressed in the brain may constitute peripheral biomarkers of CNS diseases [31,32].

Chronic stress-derived diseases are complex disorders, thus a unique biomarker (e.g., M6a) would result insufficient to cover for all the molecular and cellular cascades affected. Therefore, we aimed to search for other proteins that could help detect and monitor stress related disorders. It has already been proposed that proteins expressed in the brain may constitute peripheral biomarkers of CNS diseases [31,32]. Then the proteomic analysis of bulk serum Evs was carried out to find differentially expressed proteins potentially useful as markers of stress exposure and sensitivity. We found 36 proteins with differential expression among the groups. These results show that specific stress conditions (RIS here) modify the protein abundances in serum Evs. As shown, 60% of the proteins (COL4A2, COL6A2, CSF1R, CLU, DDI2, DPP6, FAT2, GGA1, HBA, HBB1, ITIH2, IRS2, JPH1, NOMO1, PDZD8, PRDX2, SNAP23, SLC25A20, SYN2, TENM2, USP19) express in the brain. Notably, many of the human orthologues have been associated with nervous system diseases (ADAMTSL4, CCDC171, CD2D3, COL4A2, COL6A2, CSF1R, CLU, DPP6, FCN1, HBA1, HBB1, ITIH2, IRS2, JPH, MRVI1 PRDX2, SLC25A20, SYN2, ZNF367) (S1 Table). Interestingly, a recent work showed that COL6A2 is upregulated in pain-exposed animals classified as susceptible compared with pain resilient animals [45]. This work also reported changes in MLF1, HBA, HBB and CCDC171 levels similar to those reported here (see Supplementary file, sheet 8). Although these authors used a different animal model and a different stress paradigm than us, the shared molecules suggest a certain degree of conservation in the susceptibility and/or resilience pathways. Also, DPP6 and SYN2 [46] were identified as interactors of the M6a extracellular domains, supporting the idea of a biomarker panel including M6a and its partner proteins. M6a was detected only in the control and less susceptible to stress Q3 groups and not in the Q4 (RIS) group. This may be explained because the group classification was based on M6a hippocampal levels which were downregulated by stress and, as we have previously demonstrated, correlate with M6a serum levels [4]. While many proteins identified are related to coagulation and immune system suggesting a cell blood origin for Evs, most proteins express in different tissues and organs (see S1 Table) such as liver, kidney and even testis and ovary, which points out that serum Evs come from very different cellular sources. Likewise, we have previously shown that different EV populations (i.e., with different protein content) can be found among serum Evs [4]. Furthermore, human orthologues of many proteins found in this analysis (or their isoforms) can also be detected in saliva samples [13]. This is very interesting as these molecules could be evaluated in human cohorts as a noninvasive diagnostic method for psychiatric disorders.

In addition to the differentially expressed proteins among groups, special mention should be made to those unique proteins per condition(see S1 File, sheet 7). In bulk serum Evs isolated from control animals, we found, among others, the protein 14-3-3 zeta/delta protein (YWHAZ) implicated in a variety of signaling pathways and neuronal development. YWHAZ is involved in neurogenesis, neuronal migration [47] and in the establishment of neuronal connections [48]. Knockout mice for YWHAZ present behavioral and cognitive alterations (hyperactivity, impaired memory) similar to those observed in patients affected by schizophrenia, autism and other psychiatric disorders [49]. In humans, genetic studies have associated *YWHAZ* polymorphisms with major depression and schizophrenia [50,51]. The role of the YWHAZ protein in nervous system function and its presence in the Evs of control animals make it worth investigating it as a serum biomarker for such diseases.

In RIS animals only, we detected retinol binding protein 4 RET4 (RBP4 in human) and the band 3 anion transport protein B3AT (SLC4A1, in human).A recent report showed that serum levels of RBP4 were lower in patients with MDD than that in healthy individuals and correlated with the duration of disease, which might be related to the MDD prognosis [52]. On the other hand, SLC4A1 has been associated with Parkinson’s disease and Lewy body dementia [53].

Proteins found only in the stress group (RIS) or only in the less susceptible to stress (Q3 group) and their associated molecular pathways- could also be interesting candidates for discriminating between control and RIS animals e.g., as biomarker of stress exposure or could shed light on putative pathways related to resilience or reduced sensitivity to stress. In both, RIS and less susceptible to stress Q3 groups we found proteasome-related proteins (PSMA6 and PMSA7) and components of the triglyceride lipoproteins APOC3 (Apolipoprotein C-III) and CES1D (Carboxylesterase 1D). Interestingly, the ubiquitin–proteasome system has been identified as a canonical pathway associated with neuropsychiatric disorders such as Alzheimer’s disease, psychosis and bipolar disorder [54]. The identification of CES1D and APOC3 suggests a link with lipid metabolism. This is not surprising since metabolic diseases are frequently comorbid with mental illness, although the mechanism of this association is unknown. Moreover, an increasing number of studies indicate that metabolic dysfunction and insufficient energy supply to neurons may participate in the pathogenesis of depression [55,56]. Therefore, proteins associated with metabolism such as the candidates found here can also be helpful to diagnose or monitor neuropsychiatric patients.

Interestingly, several recentre ports have related the Focal Adhesion -PI3K-Akt-mTOR-signaling pathway, the one predicted by String analysis of the candidate proteins, with the pathogenesis of depression. Li et. Al. (2023) used four depressive-like models in mice and found that the most prominent and connected path ways were PI3K/AKT, MAPK and mTOR signalling pathways [57] and Ou et. Al. (2024) found that he senet works were associated with response to lithium, the gold standard treatment for bipolar disorder [58]. Altogether this suggests that the Focal Adhesion -PI3K-Akt-mTOR-signaling pathway and its related molecules are worth further study in samples of depressed patients.

A quantifiable neuropsychiatric disease biomarker in blood or saliva that reflects the state of the brain should ideally come from the brain. Therefore, we wondered if serum M6a came, at least in part, from the brain. To test this, we used the in-utero electroporation strategy to selectively overexpress M6a-GFP in brain neurons to later evaluate M6a presence in the periphery. The neuronal promoter arranted specific neuronal expression further confirmed by no expression of M6a-GFP in GFAP positive cells. As M6a is an integral membrane protein with four transmembrane domains, it is unlikely that it could be circulating free in serum. In this sense, several of our previous publications demonstrated that M6a circulates coupled to vesicles (isolated with the same methodology used for this report). Therefore, the possibility that M6a signal could derive from other extracellular source other than Evs is low. So, with this strategy we can assert that part of serum M6a derives from brain neurons. Despite, we cannot rule out that part of the recombinant protein M6a-GFP detected in bulk serum Evs may derive from neurons located outside of the brain, e.g., neurons from the enteric nervous system, it is clear that the protein is expressed in neurons and that neuronal Evs carrying M6a are delivered to the blood. Expression in neurons outside the brain does not necessarily compromise the M6a biomarker potential, as the enteric could also be affected by stress. As suggested in Vandendriessche et al., studies comparing serum bulk Evs to the subset of M6a-carrying Evs will help to define if it is necessary to purify specific EV subsets to increase the feasibility of M6a as a biomarker[59]. The measurement of M6a in peripheral fluids as a biomarker of chronic stress offers a non-invasive and accessible method for early detection and molecular assessment. The quantification of M6a and other biomarkers might enable prompt intervention and personalized treatment strategies while advancing research on stress-related disorders for targeted public health initiatives.

We also showed, in an exploratory assay, that mice receiving Evs isolated from stressed animal serum exhibited similar phenotypes to those observed in stressed animals: increased immobility in the FST and reduced hippocampal M6a levels. Changes in the FST performance were observed 24h after EV administration, but were lost after a week. This indicates that Evs can reach brain areas other than those of the application (see S1 Fig). Once on site, Evs may induce changes on different cell groups including those that integrate those neuronal circuits underlying the FST behavior. These vesicles could transfer a set of molecules, potentially complete molecular pathways, from the stress response. This process might enable the bypassing of steps in the recipient cells, resulting in rapid effects. Moreover, such molecules could activate signaling cascades that modulate gene expression, including M6a expression. In support of this mechanism, Li et al. demonstrated that exosomal miR-207 alleviates stress symptoms in mice, possibly by transferring miRNAs from exosomes into astrocytes. In astrocytes, miR-207 binds to its target gene interactor with leucine-rich repeats (Tril) TLR4, leading to decreased release of inflammatory factors [60]. In this work, although the mechanisms by which Evs induce the observed changes are still unclear, it’s noteworthy that these changes were only observed after administration of stress-induced Evs (RIS-Evs).Therefore, we propose that RIS-Evs act as carriers of the stress signal.

In this work although the mechanisms by which Evs induce the observed changes are still unclear, it’s important to note that these changes were only observed after administration of stress-induced Evs (RIS-Evs), compared to control or PBS treatments. Therefore, we propose that RIS-Evsact as carriers of the stress signal.

Different authors reported the potential use of Evs to revert or induce depressive-like behaviors [19,20,60] and also other neurodegenerative diseases such as schizophrenia [21]. However, in none of those works, intranasal administration was used as a delivery route to obtain phenotypic changes. We chose this method because it bypasses the blood-brain barrier and may deliver Evs directly into the CNS compartment. The nasal epithelium provides an optimal absorption surface for drug delivery due to its high permeability. Moreover, the olfactory and trigeminal nerves that innervate this epithelium provide a direct route to the brain which might enhance bioavailability, limit side effects and reduce dosage [61]. Thus, our results indicate new applications for EV-mediated therapies. To further study the functional consequences of EV administration, more animals should be included as well as different doses of Evs, repetition of EV administration and a time course analysis of animals after EV administration since this would likely lead to more significant phenotypes.

Here we have shown that Evs isolated from serum of stressed animals elicit molecular and behavioral changes in recipient mice, however the study has some limitations: 1- It is still unknown the precise mechanism by which serum Evs enter into the brain and affect brain cells. Although preliminary, the biodistribution assay suggests that intranasally administered Evs can reach the brain, pass through it and spread throughout the rest of the body. This agrees with the report by Zhuang et al. (2011), where Evs could be detected in the brain at short periods post administration (3–6 h) and almost no signal was observed at 24h[62]. It has been proposed that after instillation, Evs enter the subarachnoid space and in the cerebrospinal fluid to then distribute within the brain, where they reach different brain regions, including hippocampus and cerebral cortex and enter in neurons as well as in microglial cells [63]. We do not know either the percentage of cells that have taken up Evs in the entire brain or in specific regions. To elucidate this, it would be valuable to carry out a biodistribution analysis using covalent labeling of EV proteins [64,65].2-We cannot rule out the inflammatory effects of EV administration. While our current study focused on neurobehavioral effects of EV administration, we recognize the significance of investigating inflammatory molecules in future research. Li et al. have proposed that the content of Evs derived from natural killer (NK) cells may change the expression of different cytokines pointing to neuroinflammation as a cause for neuropsychiatric diseases such as depression [60]. Conversely, Wang et al showed an effect of plasma-derived Evs independent of neuroinflammation and related to BDNF expression [19]. Although a possible reason for such differences relies on the EV source (NK cells vs. plasma), the controversy continues.3- Since we used bulk serum Evs, the cellular source of causal Evs is undefined. According to that, we have previously shown that different subsets of Evs coexist in the serum [4]. This has been confirmed with the proteomic analysis, in which many hits correspond to proteins expressed in different tissues. Furthermore, we did not address if particular EV populations are responsible for changes in mice receiving Evs, however, it seems not to be necessary as effects were observed with bulk Evs. 4- Bulk Evs could carry serum proteins/peptides on their surface that could be transferred from stressed mice to unstressed ones in addition to the EV internal cargo. Separation of non-vesicular entities from Evs is not fully achieved by common Evs isolation protocols and therefore Evs may be mixed with certain amount of free proteins, ribonucleoproteins and lipoproteins. For example, samples analyzed here do contain ApoA1, ApoB and albumin. However, as presented in the semi-quantitative data provided by the identification score -10log(p) (S1 File and S1 Table in S3E Fig), we have found no differences in the relative quantity of these proteins among groups. As a result, we can state that the observed changes attributed to the EV treatment are not influenced by these specific proteins. In addition, the contribution of other EV cargos such as microRNAs should be considered. For example, Evs isolated from serum contain miRNAs that can contribute to neurogenesis and neural plasticity, e.g., miR-133b [66], which is of particular interest since it is a regulator of M6a [8]. 5-We cannot assure the EV number administered. It is general believed that cells under stress increase exosome secretion. For example the number and the size of Evs found in plasma samples of Alzheimer’s patients have been reported to be either equal or increased in comparison to controls [59]. On the other hand, a recent work shows that chronic stress indeed increases the number of Evs but this was accompanied by protein levels [67].This indicate that chronic stress might alter the EV biogenesis and secretion, thus to avoid the administration of different amounts of Evs to control and RIS animals, to standardize, we administrated equal protein quantities. In addition, in resistance exercise sessions in which hormonal stress changes occur, a sexual dimorphism in EV content has been reported [68]. Such dimorphic Evs could later affect man and woman health and performance outcomes in a different manner. Therefore, in future works, the effects of chronic stress (RIS) on serum EV content between males and females should be compared.

Nevertheless, neither of these limitations reduces the extent of our findings since Evs isolated from RIS animals induced specific consequences not achieved by administration of control Evs. It is essential to note that the animals underwent no other manipulations apart from the EV administration and no similar changes were observed when PBS or control Evs were administered. Thus, our results strongly indicate that the effects observed in behavior and gene expression are directly attributable to the RIS-EV administration. Altogether this work aims to highlight the role of Evs as stress-signal messengers. Furthermore, we propose the intranasal administration as a non invasive brain targeting route and moreover, as a way to administer Evs loaded with candidate proteins (e.g., M6a) or with drugs for future therapeutic approaches.

## Supporting information

S1 Fig Biodistribution of serum derived extracellular vesicles (EVs) administered via intranasal. Representative images of healthy mice or brains from healthy mice 24 h after intranasal administration of either PBS (background control), boiled EVs, DiR alone or DiR-labeled EVs (suspended in PBS) from both treatments, Control and RIS. A) Whole animals were scanned face up (above) and face down (below). B) Scans of sagittal brain cuts. Color scale was set using ImageJ software. C) Scans of serum samples taken from instilled animals. Arrows indicate where the signal is observed.
(TIF)

S2 Fig Correlation between M6a values and the time spent immobile in the forced swim test (FST). A) 24h after EVs administration. B) 1 week after EVs administration.
(TIF)

S3 Fig A) Western blot of EV accepted markers: positive: Flotillin-1, CD9 and CD63 and the negative marker calnexin. B-D) uncropped, unprocessed full scans of Western blots acquired with three different scanning intensities. Highlighted with red rectangles are the parts cropped for the final image. Prior to antibody hybridization membranes were cut vertically to allow the use of different antibodies as shown by the dashed lines. E) Table shows the semi- quantitative data provided by the identification score -10log(p) after LC-MS/MS analysis. Although EV samples contain ApoA1, ApoB and albumin, there are no differences in their relative quantity between treatments and therefore we can state that the changes observed due to the EV treatment are not caused by these proteins.
(TIF)

S4 Fig Uncropped, unprocessed full scan of the Western blot from the corresponding cropped blot shown in Fig 1D. A-C different exposure times. In red and blue squares are highlighted the parts that were used to build the final blot as shown in Fig 1D. D, Western blot image as presented in Fig 1D. Molecular weight markers are indicated. Each lane represents one individual, n = 5/group. An empty lane (E) was left between control and RIS animals. Equal amounts of proteins were loaded per lane. Tubulin levels were used to normalize protein load. Prior to antibody hybridization membranes were cut at 40kDa using the Molecular Weight marker as reference as indicated by the dashed line.
(TIF)

S5 Fig Uncropped, unprocessed full scans of the Western blot shown in Fig 4C. A-C are different exposure times. D. Blot displayed in Fig 4C. The part of the blot shown for the final image is indicated in A with an orange rectangle. E Total protein staining corresponding to the Western blot in Fig 4C. All lanes were loaded with the same amount of protein. No-stain protein labeling reagent (Invitrogen) was used. NE non electroporated, 1–5 serum EVs isolated from 5 electroporated individuals.
(TIF)

S6 Fig Uncropped, unprocessed full scan of the Western blot from the corresponding cropped blot shown in Fig 5D. A-C different exposure times. D) overexposed image to further show membrane edges for the upper part. Dashed line indicates where the membrane was cut (see below). E) image as shown in Fig 5D, highlighted with a red and green rectangle, the parts cropped for the final image. Molecular weight markers are indicated. Each lane represents one individual, n = 5/group except for the PBS group where n = 4. Equal amounts of proteins were loaded per lane. Tubulin levels were used to normalize protein load. Prior to antibody hybridization membranes were cut at 40kDa using the Molecular Weight marker as reference as indicated by the dashed line.
(TIF)

S1 Table Proteins differentially expressed among groups: RIS, Q3 and control. (PDF)

S1 File Proteomic analysis raw and curated data (excluding keratins and immunoglobulin chains) for each group. RIS, Q3 and control sheets 1–6. Sheet7: Unique proteins identified in each experimental group. Panther analysis of molecular pathways. Sheet 8: Differentially expressed proteins among experimental groups. SynGO data for identification of synaptic proteins is also shown. Sheet 9: Differentially expressed proteins among all three experimental groups.Sheet 10: String analysis of the 21 candidates previously identified in the brain and/or brain EVs.
(XLSX)

S2 File Raw data for graphics on Figs 1–5. (XLSX)
