# Supplementary figures and images for: Bulk serum extracellular vesicles from stressed mice show a distinct proteome and induce behavioral and molecular changes in naive mice
Source: PLoS One. 2024 Aug 15;19(8):e0308976. doi: 10.1371/journal.pone.0308976 (PMC11326636; doi:10.1371/journal.pone.0308976)

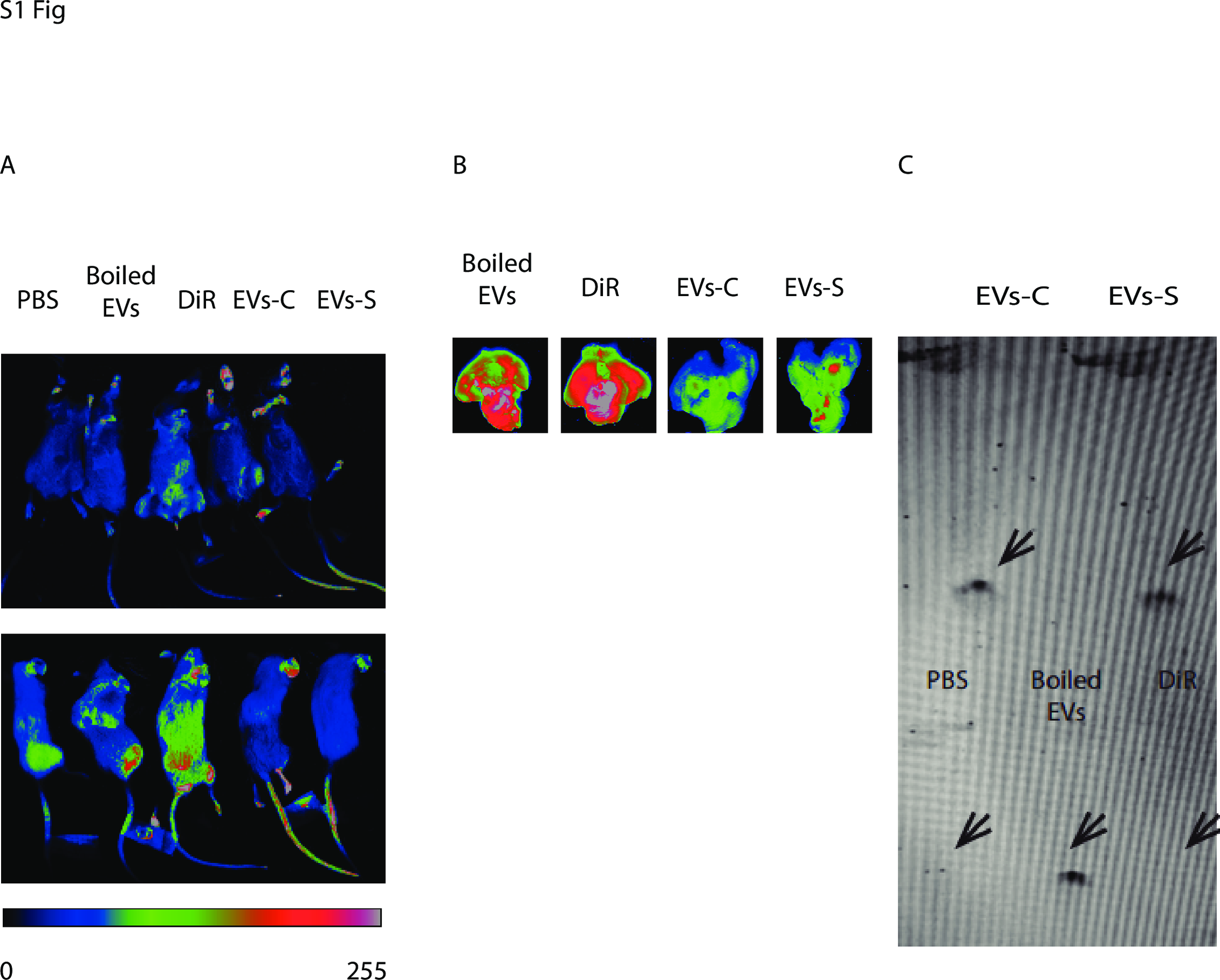

Supplement: S1 Fig — Representative images of healthy mice or brains from healthy mice 24 h after intranasal administration of either PBS (background control), boiled EVs, DiR alone or DiR-labeled EVs (suspended in PBS) from both treatments, Control and RIS. A) Whole animals were scanned face up (above) and face down (below). B) Scans of sagittal brain cuts. Color scale was set using ImageJ software. C) Scans of serum samples taken from instilled animals. Arrows indicate where the signal is observed. (TIF) [file pone.0308976.s001.tif]

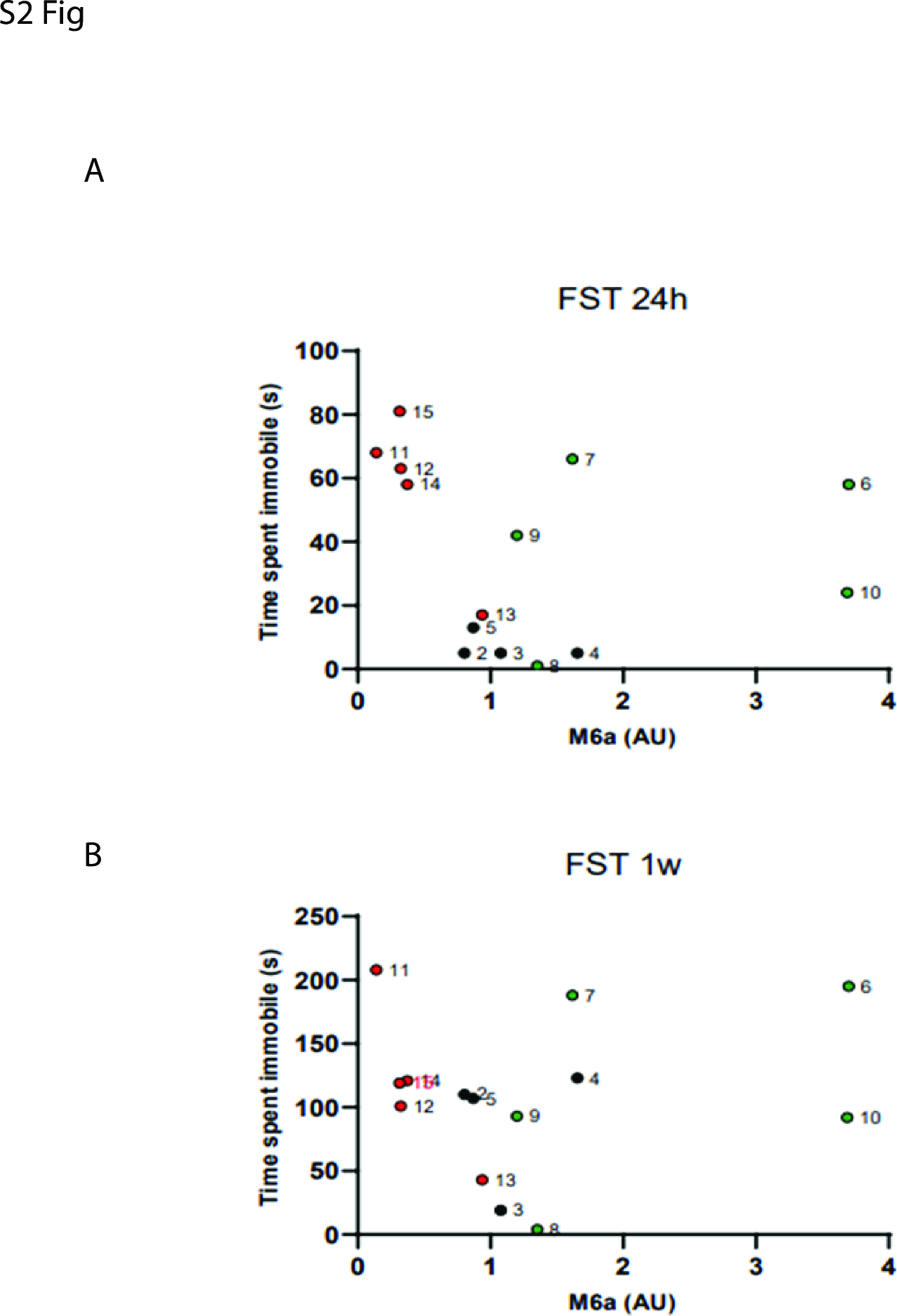

Supplement: S2 Fig — A) 24h after EVs administration. B) 1 week after EVs administration. (TIF) [file pone.0308976.s002.tif]

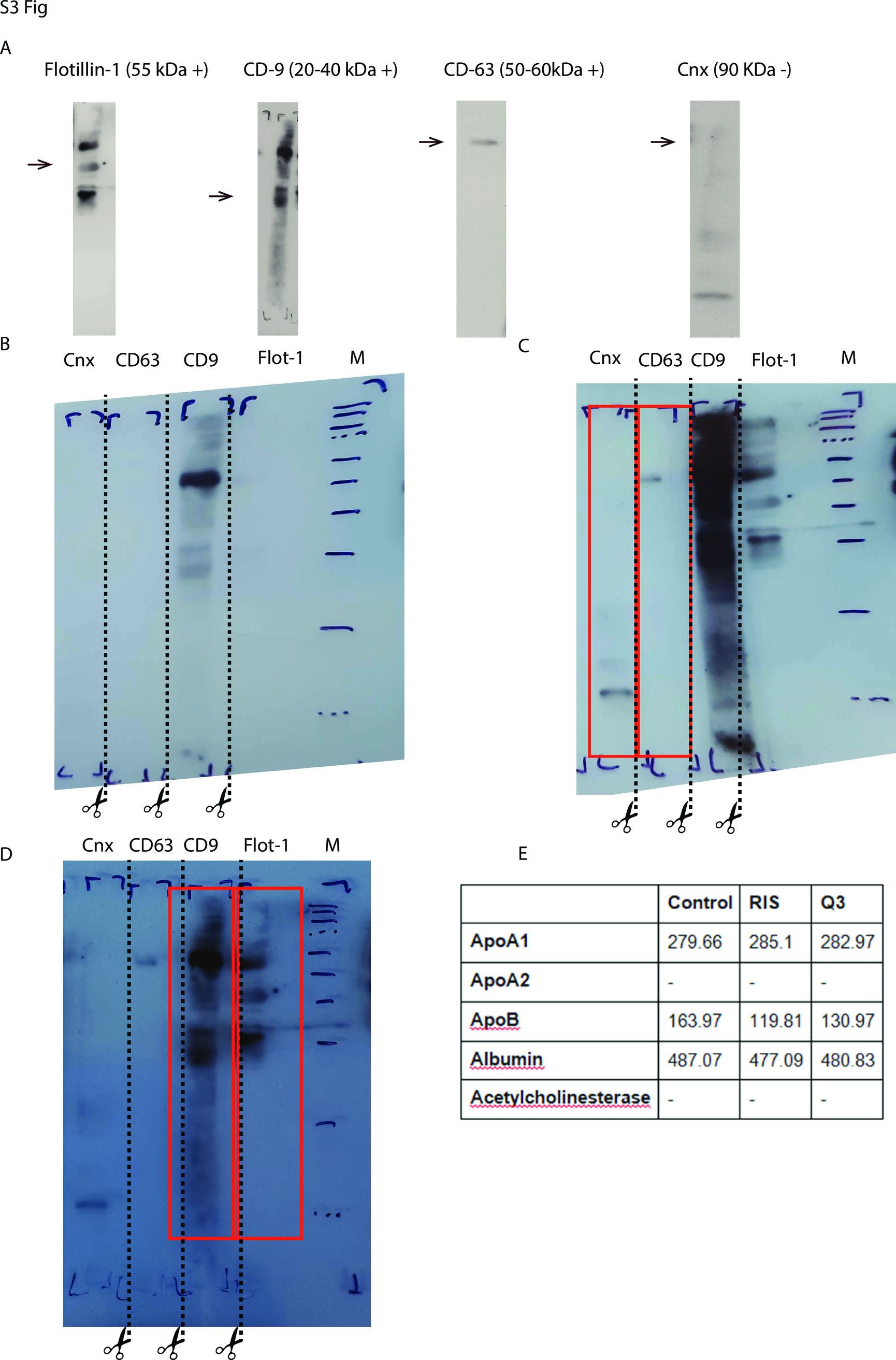

Supplement: S3 Fig — A) Western blot of EV accepted markers: positive: Flotillin-1, CD9 and CD63 and the negative marker calnexin. B-D) uncropped, unprocessed full scans of Western blots acquired with three different scanning intensities. Highlighted with red rectangles are the parts cropped for the final image. Prior to antibody hybridization membranes were cut vertically to allow the use of different antibodies as shown by the dashed lines. E) Table shows the semi- quantitative data provided by the identification score -10log(p) after LC-MS/MS analysis. Although EV samples contain ApoA1, ApoB and albumin, there are no differences in their relative quantity between treatments and therefore we can state that the changes observed due to the EV treatment are not caused by these proteins. (TIF) [file pone.0308976.s003.tif]

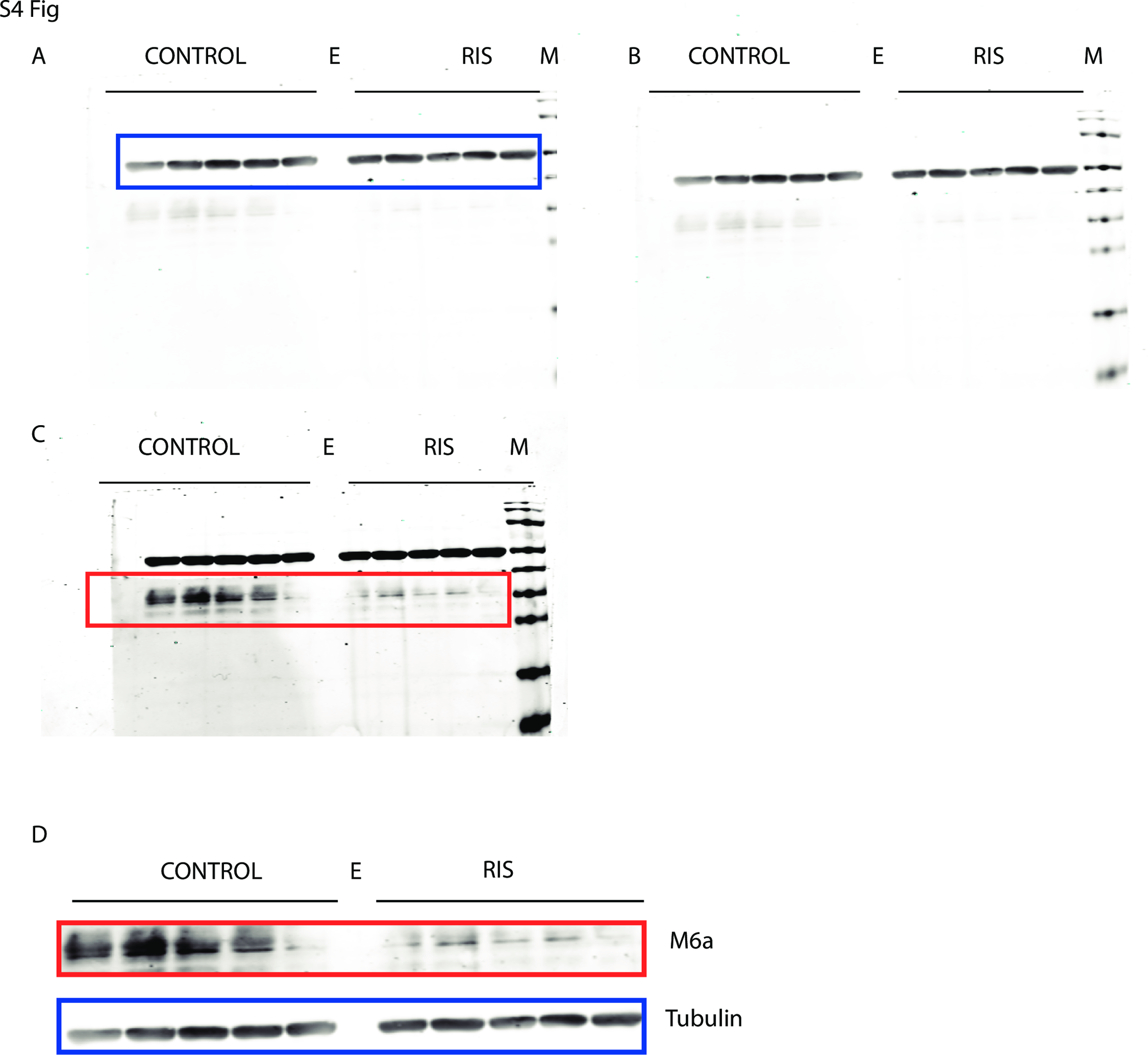

Supplement: S4 Fig — A-C different exposure times. In red and blue squares are highlighted the parts that were used to build the final blot as shown in Fig 1D. D, Western blot image as presented in Fig 1D. Molecular weight markers are indicated. Each lane represents one individual, n = 5/group. An empty lane (E) was left between control and RIS animals. Equal amounts of proteins were loaded per lane. Tubulin levels were used to normalize protein load. Prior to antibody hybridization membranes were cut at 40kDa using the Molecular Weight marker as reference as indicated by the dashed line. (TIF) [file pone.0308976.s004.tif]

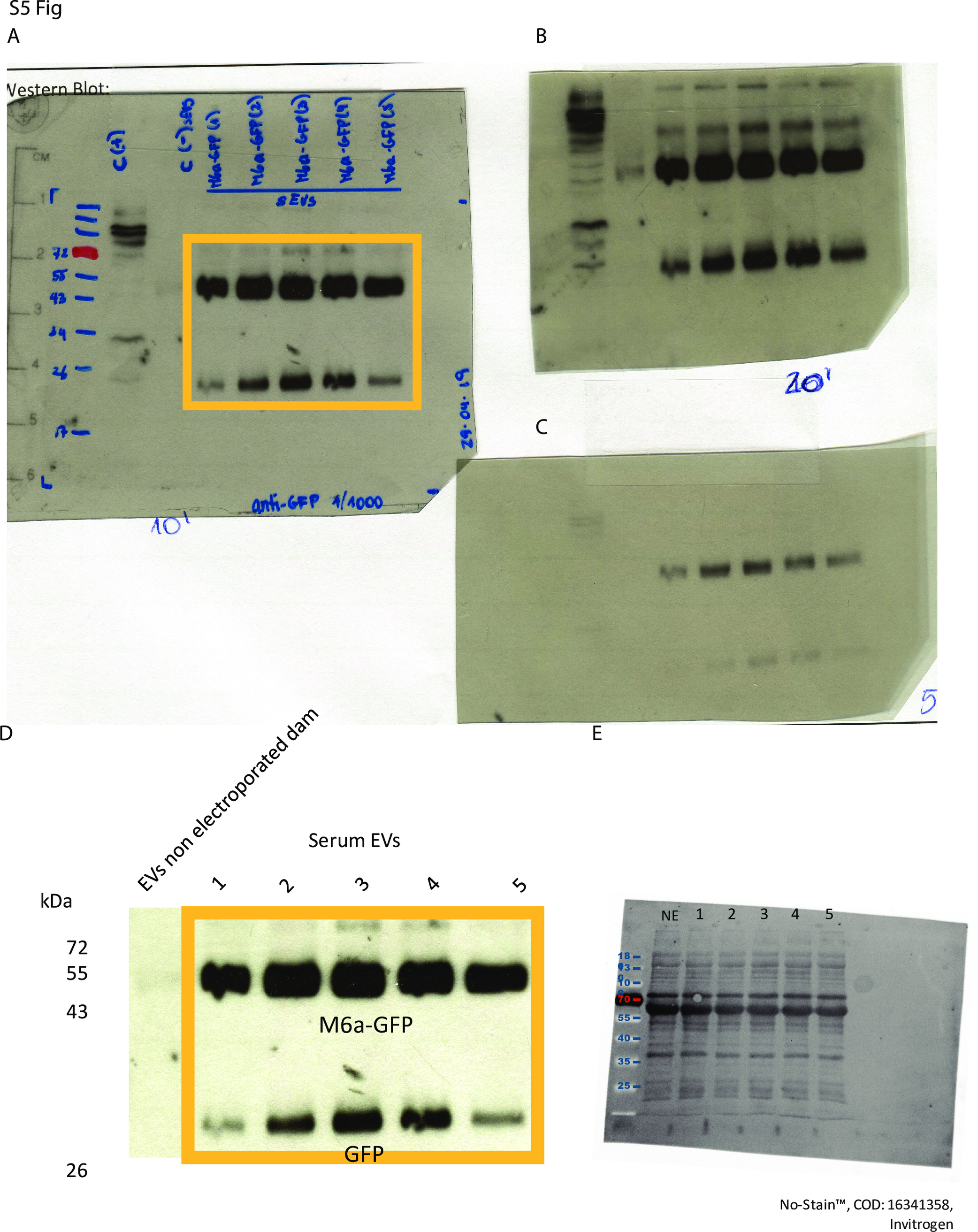

Supplement: S5 Fig — A-C are different exposure times. D. Blot displayed in Fig 4C. The part of the blot shown for the final image is indicated in A with an orange rectangle. E Total protein staining corresponding to the Western blot in Fig 4C. All lanes were loaded with the same amount of protein. No-stain protein labeling reagent (Invitrogen) was used. NE non electroporated, 1–5 serum EVs isolated from 5 electroporated individuals. (TIF) [file pone.0308976.s005.tif]

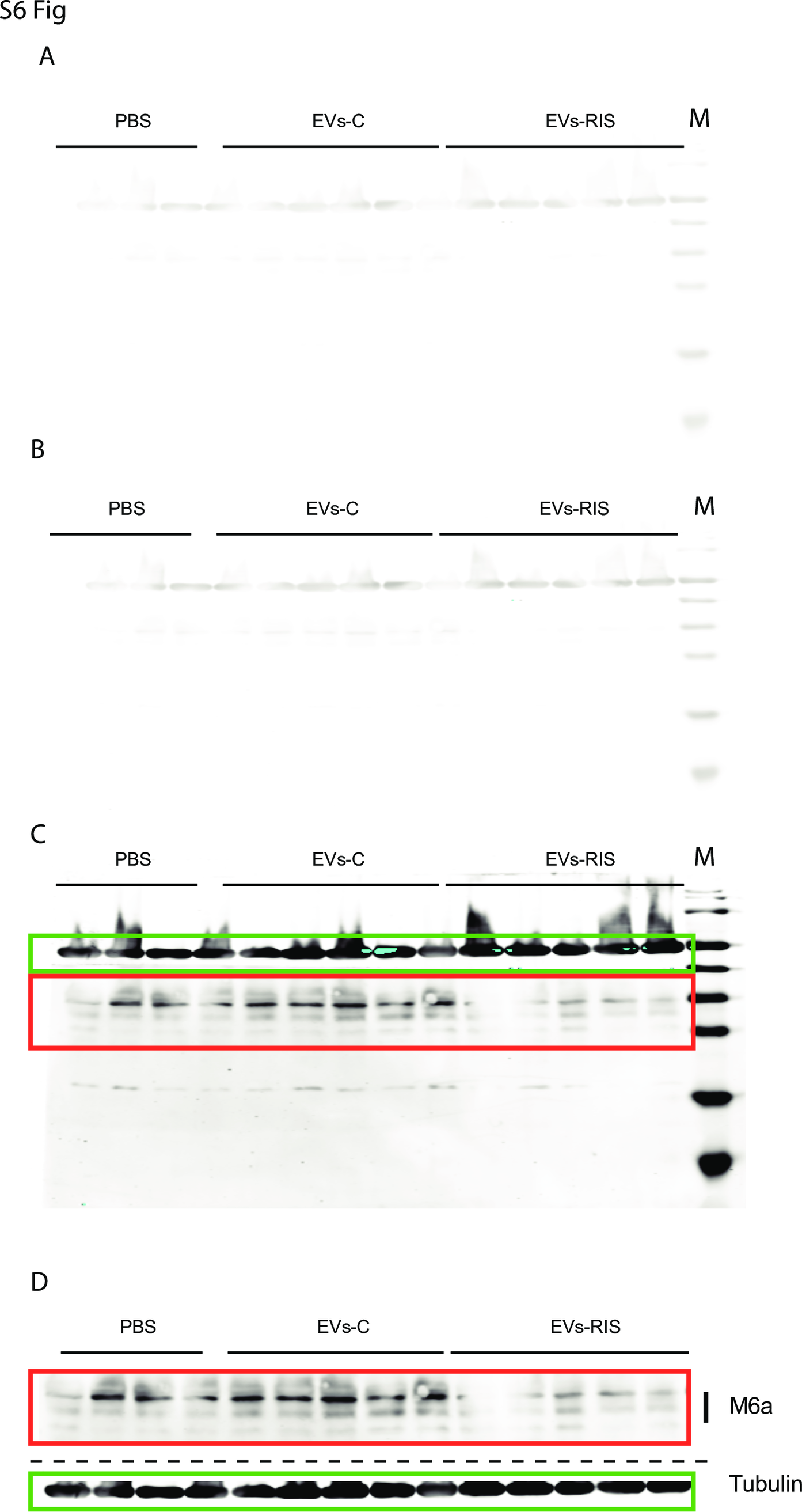

Supplement: S6 Fig — A-C different exposure times. D) overexposed image to further show membrane edges for the upper part. Dashed line indicates where the membrane was cut (see below). E) image as shown in Fig 5D, highlighted with a red and green rectangle, the parts cropped for the final image. Molecular weight markers are indicated. Each lane represents one individual, n = 5/group except for the PBS group where n = 4. Equal amounts of proteins were loaded per lane. Tubulin levels were used to normalize protein load. Prior to antibody hybridization membranes were cut at 40kDa using the Molecular Weight marker as reference as indicated by the dashed line. (TIF) [file pone.0308976.s006.tif]
