# Supplementary material for: Bulk serum extracellular vesicles from stressed mice show a distinct proteome and induce behavioral and molecular changes in naive mice
Source: PLoS One. 2024 Aug 15;19(8):e0308976. doi: 10.1371/journal.pone.0308976 (PMC11326636; doi:10.1371/journal.pone.0308976)
Supplement: S1 Table — (PDF) [file pone.0308976.s007.pdf]

Supplementary Table 1. Proteins differentially expressed among groups: RIS, Q3 and control

Accession number	Protein	Association with nervous system disorders		Expression
		**Bibliography [a]**	**Malacards [b]**	
Q80T21	Adamtsl4<br>ADAMTS-like protein 4	Primary Glioblastoma Multiforme [1]<br>Migraine and cervical artery dissection [2]	Craniosynostosis with Ectopia Lentis *<br>Bilateral Frontal Polymicrogyria ‡<br>Glioma susceptibility ‡	Widely expressed in a range of tissues. Especially prevalent in the brain, spinal cord, muscle, lung, heart, liver, bone marrow.
Q52KB6	C2CD3<br>C2 Domain Containing 3 Centriole Elongation Regulator	AD [3]	Orofaciodigital Syndrome *<br>Joubert Syndrome *<br>Microcephaly §<br>Meckel Syndrome §<br>Cone-Rod Dystrophy §<br>Coach Syndrome ‡<br>Spinocerebellar Ataxia ‡<br>Retinitis pigmentosa ‡<br>Spina Bifida Meningocele ‡<br>Congenital Nervous System Abnormality ‡<br>Neural Tube Defects ‡	Widely expressed in a range of tissues/organs including eye, nervous system, bone, bone marrow, gut
E9Q1U1	Ccdc171<br>Coiled-coil domain-containing protein 171	Potential genetic risk factor for BD [4]	N/A	Overexpressed in the testis, lung, brain and pancreas.
Q8C6S9	Cfap54<br>Cilia- and flagella-associated protein 54	Hydrocephalus [5]<br>ALS [6]	Hydrocephalus ‡	Overexpressed in cervix. Expressed in the brain, breast, colon
Q06890	CLU<br>Clusterin	AD [7]<br>TBI and stroke [8]<br>Alpha-synucleopathies and PD [9]	Familial AD §<br>Mild Cognitive Impairment §<br>Dementia §<br>Creutzfeldt-	Widely expressed in a range of tissues/organs including

			Jakob Disease § Scrapie § Cerebral Amyloid Angiopathy, Cst3-Related ‡ Macular Degeneration ‡ Normal Pressure Hydrocephalus ‡ Pick Disease of Brain ‡ Retinitis Pigmentosa ‡ Congenital Disorder of Glycosylation ‡ ALS ‡ PD ‡ Nervous System Disease ‡ Wallerian Degeneration ‡ Neuroblastoma ‡	nervous system, liver, eye, intestine, testis, ovary, heart, kidney blood, vitreous humor, synovial fluid and cerebrospinal fluid
P08122	Col4a2 Collagen type 4 alpha-2 chain	Neurological abnormalities, including cortical development [10]	Brain Small Vessel Disease * Intracerebral hemorrhage * Porencephaly * Cerebral Palsy § Optic Nerve Hypoplasia § Schizencephaly ‡ Polymicrogyria ‡	Widely expressed in a range of tissues/organs including eye, skin, liver, nervous system and pancreas
Q02788	Col6a2 Collagen type 6 alpha-2 chain	Potential neuroprotective for AD [11]	Down syndrome § Progressive Myoclonus Epilepsy § Walker-Warburg Syndrome ‡ Mitochondrial complex I deficiency ‡ Neurogenic Bladder ‡ Spasticity ‡ Glioma ‡	Widely expressed in a range of tissues/organs including lung, liver, bone and nervous system.
P09581	Csf1r Macrophage colony-stimulating factor 1 receptor	Pathophysiology of MDD [12] CSF1R mutation carriers may present clinical phenotypes of AD, FTD, PD, and MS, accompanied with	Leukoencephalopathy * Brain Abnormalities, Neurodegeneration and Dysosteosclerosis* Csf1r-Related	Widely expressed in a range of tissues/organs including nervous system, bone

		white matter abnormalities [13]	Adult-Onset Leukoencephalopathy with Axonal Spheroids and Pigmented Glia *<br>Familial AD §<br>Dementia §<br>Frontotemporal dementia §<br>Primary Progressive MS §<br>Cerebral degeneration ‡<br>Parkinsonism ‡<br>Spasticity ‡<br>Glioblastoma ‡<br>Demyelinating disease ‡<br>Neuroaxonal Dystrophy ‡<br>Encephalopathy ‡<br>Aphasia ‡	marrow, liver, bone and spleen.
A2ADY9	Ddi2<br>Protein DDI1 homolog 2	N/A	Ngly1-Deficiency ‡<br>Combat Disorder ‡	Widely expressed in a range of tissues/organs including nervous system, peripheral blood mononuclear cells, lymph node, skeletal muscle
Q9Z218	Dpp6<br>Dipeptidyl aminopeptidase-like protein 6	Loss impairs learning and memory and affects neurodevelopment [14]<br>Dementia [15]	Intellectual developmental disorder *<br>Microcephaly *<br>ALS §<br>SCZ §<br>ASD §<br>Tourette syndrome §<br>Stiff-Person Syndrome ‡<br>La Crosse Encephalitis ‡<br>Vulto-Van Silfhout-De Vries Syndrome ‡<br>Hyperekplexia ‡	Highly expressed in the brain cortex. Also found in the pancreas, muscle, eye, thyroid gland.

			Choreatic Disease ‡<br>Jawad Syndrome ‡<br>Neuroblastoma ‡<br>Frontotemporal Dementia ‡<br>Tardive Dyskinesia ‡<br>Dementia ‡<br>Aging ‡<br>Learning Disability ‡<br>Tic Disorder ‡<br>Cerebellar Atrophy, Developmental Delay and Seizures ‡	
Q5F226	Fat2<br>Protocadherin Fat 2	ASD [16]<br>Requirement for hypothalamic-pituitary development [17]	Spinocerebellar Ataxia *<br>Spastic Paraplegia ‡<br>Cerebellar Ataxia ‡<br>Stocco Dos Santos Type X-Linked Intellectual Disability ‡<br>Spastic Ataxia ‡<br>Meningioma ‡,<br>Neuronal Migration Disorders ‡<br>Brain Cancer ‡<br>Retinitis Pigmentosa ‡<br>Neurofibromatosis ‡<br>Acoustic Neuroma ‡	Detected in brain, gut tube, neural tube and bone
O70165	FCN1<br>Ficolin 1	Cerebral Ischemia [18]<br>TBI [19]	Leprosy ‡<br>3mc Syndrome ‡<br>Ataxia with vitamin E deficiency ‡	Peripheral blood leukocytes, monocytes and granulocytes
Q8R0H9	Gga1<br>ADP-ribosylation factor-binding protein GGA1	AD pathogenesis [20]	Chronic Bilirubin Encephalopathy ‡<br>Familial AD ‡<br>TBI ‡<br>Niemann-Pick Disease ‡	Widely expressed in a range of tissues/organs including

				nervous system, liver, heart and kidney.
P01942<br><br>P02088	HBA1 Hemoglobin subunit alpha<br><br>HBB Hemoglobin subunit beta-1	HBA is used for discriminating MDD from BD [21] HBA1 and HBB associated with Neurodegenerative Diseases [22] Altered in MDD individuals [23] Potential markers of chronic social stress in mice [24] Markers in AD [25]	HBA1 Alpha Thalassemia-Intellectual Disability Syndrome Type 1 §<br><br>HBB Retinitis Pigmentosa ‡ Microcephaly ‡ Friedreich Ataxia ‡ Down Syndrome ‡ Noonan syndrome ‡ Neuroblastoma ‡	HBA1 Widely expressed in a range of tissues/organs including heart, spleen, liver, bone marrow, blood, nervous system and lung<br><br>HBB Widely expressed including cerebral cortex
P81122	Irs2 Insulin receptor substrate 2	Gene variant associated with cognitive impairment [26] Association with SCZ and BD [27] Association with refractory temporal lobe epilepsy [28]	Growth delay due to IGF-I resistance* Retinitis Pigmentosa ‡ Ataxia-Telangiectasia ‡ Telangiectasis ‡ Glioma Susceptibility ‡ Convulsions ‡ HD ‡ Neuroblastoma ‡	Skeletal muscle, lung, brain, liver, kidney, heart and spleen.
Q61703	ITIH2 inter-alpha-trypsin inhibitor	MS [29] Useful for discriminating MDD from BD [21] Reduced in MDD [30]	N/A	Liver, brain, ovary, pancreas, spleen, bone marrow.
Q9ET80	JPH Junctophilin 1	Associated with various forms of Charcot-Marie-Tooth Disease and neuropathy [31]	Charcot-Marie-Tooth Disease* HD ‡ Malignant hyperthermia ‡	Widely expressed in a range of tissues/organs

				including skeletal muscle, heart, frontal cortex, gallbladder, pancreas.
Q5DTI6	KANS1L1<br>KAT8 Regulatory NSL Complex Subunit 1	Anxiety [32]<br>MDD [33]<br>Attention-deficit hyperactivity disorder (ADHD) [34]<br>PD [35]<br>PTSD [36]<br>Hippocampal atrophy [37]	Koolen-De Vries Syndrome*<br>Epilepsy §<br>Chromosome 17q21.31 Duplication Syndrome ‡<br>Supranuclear Palsy ‡<br>Mowat-Wilson Syndrome ‡<br>Pitt-Hopkins Syndrome ‡<br>Kleefstra ‡ Syndrome ‡<br>Christianson Syndrome ‡<br>Williams-Beuren Syndrome ‡<br>Syndromic Intellectual Disability ‡<br>Hypotonia ‡<br>Apraxia ‡<br>Agnosia ‡<br>Speech Disorder ‡<br>Agenesis of Corpus Callosum ‡<br>Craniosynostosis ‡<br>Optic Nerve Disease ‡	Widely expressed in a range of tissues/organs including nervous system, liver, blood, muscle, thyroid gland, kidney.
Q6PCM1	Kdm3a<br>Lysine-specific demethylase 3A	Neuronal development [38]	Microphthalmia ‡	Widely expressed in a range of tissues/organs including brain, breast, lung, blood, lung, muscle, bone marrow.
Q9WUX5	Mrvi1/<br>Protein MRVI1/<br>Inositol 1,4,5-triphosphate receptor	Associated with lacunar stroke [39]<br>Associated with cerebrovascular disorders [2]	Moyamoya Disease ‡<br>Retinitis Pigmentosa ‡<br>Glioma	Widely expressed in a range of tissues/organs including

	associated 1		Susceptibility ‡<br>Oligodendroglioma ‡<br>Astrocytoma ‡<br>Tyrosinemia ‡	nervous system, liver, blood, muscle, lymph nodes.
Q9QWV4	Mlf1<br>Myeloid leukemia factor 1	Susceptibility genes for ASD [40]<br>In a rat model, associated to alcohol consumption [41]	Laurence-Moon Syndrome §<br>Chronic Bilirubin Encephalopathy ‡<br>HD ‡<br>Neuroblastoma ‡	Widely expressed in a range of tissues/organs including nervous system, testis, ovary, heart.
Q6GQT9	Nomo1<br>Nodal modulator 1	Glioma susceptibility gene [42]	N/A	Widely expressed in a range of tissues/organs including pancreas, frontal cortex, liver, lung.
B9EJ80	Pdzd8<br>PDZ domain-containing protein 8	Associated with PTSD [43]<br>Risk of intellectual disability [44]	ASD ‡	Widely expressed in a range of tissues/organs including nervous system, liver, bone marrow, pancreas.
Q61171	PRDX2<br>Peroxiredoxin-2	Useful for discriminating MDD from BD and from SCZ [21]<br>MS [45]<br>PD [46]<br>ALS [47]<br>AD [48]<br>In a rat model, associated with stress resilience [49]<br>Acute intracerebral hemorrhage [50]	Down Syndrome ‡<br>Neuroblastoma ‡	Widely expressed
Q8CGM2	Rp1l1<br>Retinitis pigmentosa 1-	Ocular anomalies of 8p23.1 duplication	Retinitis pigmentosa*<br>Fundus dystrophy	Retinal-specific. Also expressed in

	like 1 protein	syndrome (characterized by developmental delays and/or learning difficulties) [51]<br>Associated to brain arteriovenous malformation [52]	§<br>Macular degeneration §<br>Several eye diseases ‡	neurons and heart.
Q9Z2Z6	Slc25a20 Mitochondrial carnitine/ acylcarnitine carrier protein	Potential role in acylcarnitine's antidepressant effects [53]<br>ALS [54]	Carnitine-acylcarnitine translocase (CACT) deficiency *<br>Mitochondrial complex I deficiency §<br>Combined D-2- and L-2-Hydroxyglutaric Aciduria §<br>Gait apraxia §	Widely expressed in a range of tissues/organs including liver, gallbladder, heart, testis, brain.
O09044	Snap23 Synaptosomal-associated protein 23	SCZ [55]<br>Brain development [56]	Cerebral Dysgenesis, Neuropathy, Ichthyosis, and Palmoplantar Keratoderma Syndrome (CEDNIK) ‡<br>Chronic Bilirubin Encephalopathy ‡	Widely expressed in a range of tissues/organs including blood, liver, nervous system, spleen, kidney.
Q99MY0	Spz1 Spermatogenic leucine zipper protein	Glioma's malignant progression [57]	Glioma Susceptibility ‡	Strongly expressed in the testis and epididymis. Expressed in several tumor cell lines and modestly, in the nervous system.
Q64332	Syn2 Synapsin-2	MDD and BD [58]<br>AD [59]<br>Creutzfeldt-Jakob disease [60]<br>Alcoholism dependence [61]	SCZ *<br>BD §<br>Epilepsy §<br>ASD §<br>Psychotic Disorder §<br>Persian Gulf Syndrome ‡<br>Neuromuscular Disease ‡	Highly expressed in the central and peripheral nervous systems. Also expressed in the reproductive

				organs.
Q9WTS5	Tenm2 Teneurin-2	In mice, associated to spatial learning [62]	Glioma Susceptibility ‡ Meningioma ‡ Oligodendroglioma Astrocytoma ‡ Simpson-Golabi-Behmel Syndrome ‡	Widely expressed in a range of tissues/organs including brain, heart, liver, kidney and weakly expressed in lung and testis.
F8VPN2	Tex15 Testis-expressed protein 15	N/A	PD § Riddle Syndrome ‡	Detected in testis and ovary and at lower levels in lung and brain
Q2QI47	Ush2A Usherin	Usher syndrome patients suffer from schizophrenia-like disorder and psychotic symptoms [63]	Usher's Syndrome * Retinitis Pigmentosa * Retinal Degeneration * Late-Onset Retinal Degeneration (LORD) * Cone-Rod Dystrophy ‡ Rare Genetic Deafness § Intellectual Developmental Disorder with Language Impairment and with or Without Autistic Features ‡ Several and different inherited retinal and ear disorders ‡	Detected in retina, cochlea, small and large intestine, pancreas and at lower levels in the brain
Q3UJD6	Usp19 Ubiquitin carboxyl-terminal hydrolase 19	Association with PD [64]	Pitt-Hopkins-Like Syndrome 2 ‡ HD ‡	Widely expressed in a range of tissues/organs including nervous system, skin, muscle, kidney
Q8C2E7	Washc5	AD [65]	Spastic Paraplegia	Widely

	WASH complex subunit 5		* Ritscher-Schinzel Syndrome * PD Late-Onset § Frontotemporal Dementia § Hermansky-Pudlak Syndrome § Masa Syndrome ‡ Headache ‡	expressed in a range of tissues/organs including lung, bone marrow, nervous system, adrenal gland, kidney, liver
Q0VDT2	Znf367 Zinc finger protein 367	In brain teleost, associated to aging [66] In *Xenopus laevis,* a putative role in adult neurogenesis [67]	Down Syndrome ‡ Cone-Rod Dystrophy 2 ‡ Pheochromocytoma ‡ Paraganglioma ‡ Glioma Susceptibility ‡	Expressed in bone marrow and ovary. Also in brain, heart and testis.

a: Articles included mostly refer to disorders not included in Malacards.
b: Classification according GeneCards and MalaCards: asterisk (*) indicates elite association, § score>10.0, ‡ score >1.0<10.0 and classified in the anatomical categories: *Neuronal diseases* or *Mental diseases.* N/A: non-available, MDD: major depressive disorder, BP: bipolar disorder, SCZ: schizophrenia, PD: Parkinson's disease, AD: Alzheimer's disease, HD: Huntington's disease, ALS: Amyotrophic lateral sclerosis, MS: Multiple sclerosis, TBI: Traumatic brain injury, PTSD: Postraumatic stress disorder, ASD: Autism spectrum disorder.

References

1. Zhao Z, Zhang KN, Chai RC, et al (2019) ADAMTSL4, a secreted glycoprotein, is a novel immune-related biomarker for primary glioblastoma multiforme. Dis Markers 2019:. https://doi.org/10.1155/2019/1802620
2. Daghals I, Sargurupremraj M, Danning R, et al (2022) Migraine, Stroke, and Cervical Arterial Dissection: Shared Genetics for a Triad of Brain Disorders with Vascular Involvement. Neurol Genet 8:1–12. https://doi.org/10.1212/NXG.0000000000000653
3. Prokopenko D, Morgan SL, Mullin K, et al (2021) Whole-genome sequencing reveals new Alzheimer's disease–associated rare variants in loci related to synaptic function and neuronal development. Alzheimer's Dement 17:1509–1527. https://doi.org/10.1002/alz.12319
4. Husson T, Duboc JB, Quenez O, et al (2018) Identification of potential genetic risk factors for bipolar disorder by whole-exome sequencing. Transl Psychiatry 8:. https://doi.org/10.1038/s41398-018-0291-7
5. McKenzie CW, Preston CC, Finn R, et al (2018) Strain-specific differences in brain gene expression in a hydrocephalic mouse model with motile cilia dysfunction. Sci Rep 8:1–13. https://doi.org/10.1038/s41598-018-31743-5
6. Benito PA, Moreno J, Aso E, et al (2017) Amyotrophic lateral sclerosis, gene deregulation in the anterior. 9:823–851
7. Wu ZC, Yu JT, Li Y, Tan L (2012) Clusterin in Alzheimer's disease, 1st ed. Elsevier Inc.
8. Imhof A, Charnay Y, Vallet PG, et al (2006) Sustained astrocytic clusterin expression improves remodeling after brain ischemia. Neurobiol Dis 22:274–283. https://doi.org/10.1016/j.nbd.2005.11.009

9. Sasaki K, Doh-ura K, Wakisaka Y, Iwaki T (2002) Clusterin/apolipoprotein J is associated with cortical Lewy bodies: Immunohistochemical study in cases with α-synucleinopathies. Acta Neuropathol 104:225–230. https://doi.org/10.1007/s00401-002-0546-4
10. Neri S, Ferlazzo E, Africa E, et al (2021) Novel COL4A2 mutation causing familial malformations of cortical development. Eur Rev Med Pharmacol Sci 25:898–905. https://doi.org/10.26355/eurrev_202101_24658
11. Cheng JS, Dubal DB, Kim DH, et al (2009) Collagen VI protects neurons against Aβ toxicity. Nat Neurosci 12:119–121. https://doi.org/10.1038/nn.2240
12. Zhang J, Chang L, Pu Y, Hashimoto K (2020) Abnormal expression of colony stimulating factor 1 receptor (CSF1R) and transcription factor PU.1 (SPI1) in the spleen from patients with major psychiatric disorders: A role of brain–spleen axis. J Affect Disord 272:110–115. https://doi.org/10.1016/j.jad.2020.03.128
13. Hu B, Duan S, Wang Z, et al (2021) Insights Into the Role of CSF1R in the Central Nervous System and Neurological Disorders. Front Aging Neurosci 13:1–17. https://doi.org/10.3389/fnagi.2021.789834
14. Lin L, Murphy JG, Karlsson RM, et al (2018) DPP6 loss impacts hippocampal synaptic development and induces behavioral impairments in recognition, learning and memory. Front Cell Neurosci 12:1–14. https://doi.org/10.3389/fncel.2018.00084
15. Cacace R, Heeman B, Van Mossevelde S, et al (2019) Loss of DPP6 in neurodegenerative dementia: a genetic player in the dysfunction of neuronal excitability. Acta Neuropathol 901–918. https://doi.org/10.1007/s00401-019-01976-3
16. Butler MG, Rafi SK, Hossain W, et al (2015) Whole exome sequencing in females with autism implicates novel and candidate genes. Int J Mol Sci 16:1312–1335. https://doi.org/10.3390/ijms16011312
17. Lodge EJ, Xekouki P, Silva TS, et al (2020) Requirement of FAT and DCHS protocadherins during hypothalamic-pituitary development. JCI Insight 5:. https://doi.org/10.1172/jci.insight.134310
18. Llull L, Thiel S, Amaro S, et al (2017) Ficolin-1 Levels in Patients Developing Vasospasm and Cerebral Ischemia After Spontaneous Subarachnoid Hemorrhage. Mol Neurobiol 54:6572–6580. https://doi.org/10.1007/s12035-016-0180-0
19. Ciechanowska A, Ciapała K, Pawlik K, et al (2021) Initiators of classical and lectin complement pathways are differently engaged after traumatic brain injury—time-dependent changes in the cortex, striatum, thalamus and hippocampus in a mouse model. Int J Mol Sci 22:1–30. https://doi.org/10.3390/ijms22010045
20. Wahle T, Thal DR, Sastre M, et al (2006) GGA1 is expressed in the human brain and affects the generation of amyloid β-peptide. J Neurosci 26:12838–12846. https://doi.org/10.1523/JNEUROSCI.1982-06.2006
21. Shin D, Kim H, Yeo I, Kim J (2022) Integrating proteomic and clinical data to discriminate major psychiatric disorders : Applications for major depressive disorder , bipolar disorder , and schizophrenia. https://doi.org/10.1002/ctm2.929
22. Vanni S, Zattoni M, Moda F, et al (2018) Hemoglobin mRNA changes in the frontal cortex of patients with neurodegenerative diseases. Front Neurosci 12:1–12. https://doi.org/10.3389/fnins.2018.00008
23. Malki K, Pain O, Tosto MG, et al (2015) Identification of genes and gene pathways associated with major depressive disorder by integrative brain analysis of rat and human prefrontal cortex transcriptomes. Transl Psychiatry 5:. https://doi.org/10.1038/tp.2015.15
24. A.M. S, J. G, A.H. S, et al (2014) Social stress increases expression of hemoglobin genes in mouse prefrontal cortex. BMC Neurosci 15:130
25. Arioz BI, Tufekci KU, Olcum M, et al (2021) Proteome profiling of neuron-derived exosomes in Alzheimer's disease reveals hemoglobin as a potential biomarker. Neurosci

Lett 755:1–6. https://doi.org/10.1016/j.neulet.2021.135914
26. Mazzoccoli G, Dagostino MP, Paroni G, et al (2017) Analysis of MTNR1B gene polymorphisms in relationship with IRS2 gene variants, epicardial fat thickness, glucose homeostasis and cognitive performance in the elderly. Chronobiol Int 34:1083–1093. https://doi.org/10.1080/07420528.2017.1340894
27. Takayanagi Y, Ishizuka K, Laursen TM, et al (2021) From population to neuron: exploring common mediators for metabolic problems and mental illnesses. Mol Psychiatry 26:3931–3942. https://doi.org/10.1038/s41380-020-00939-5
28. Che F, Fu Q, Li X, et al (2015) Association of insulin receptor H1085H C>T, insulin receptor substrate 1 G972R and insulin receptor substrate 2 1057G/A polymorphisms with refractory temporal lobe epilepsy in Han Chinese. Seizure 25:178–180. https://doi.org/10.1016/j.seizure.2014.09.014
29. Mosleth EF, Vedeler CA, Liland KH, et al (2021) Cerebrospinal fluid proteome shows disrupted neuronal development in multiple sclerosis. Sci Rep 11:1–19. https://doi.org/10.1038/s41598-021-82388-w
30. Wang Q, Su X, Jiang X, et al (2016) ITRAQ technology-based identification of human peripheral serum proteins associated with depression. Neuroscience 330:291–325. https://doi.org/10.1016/j.neuroscience.2016.05.055
31. Pla-Martín D, Calpena E, Lupo V, et al (2015) Junctophilin-1 is a modifier gene of GDAP1-related Charcot-Marie-Tooth disease. Hum Mol Genet 24:213–229. https://doi.org/10.1093/hmg/ddu440
32. Su X, Li W, Lv L, et al (2021) Transcriptome-Wide Association Study Provides Insights Into the Genetic Component of Gene Expression in Anxiety. Front Genet 12:1–12. https://doi.org/10.3389/fgene.2021.740134
33. Mahajan GJ, Vallender EJ, Garrett MR, et al (2018) Altered neuro-inflammatory gene expression in hippocampus in major depressive disorder. Prog Neuro-Psychopharmacology Biol Psychiatry 82:177–186. https://doi.org/10.1016/j.pnpbp.2017.11.017
34. Fernàndez-Castillo N, Cabana-Domínguez J, Kappel DB, et al (2022) Exploring the Contribution to ADHD of Genes Involved in Mendelian Disorders Presenting with Hyperactivity and/or Inattention. Genes (Basel) 13:1–12. https://doi.org/10.3390/genes13010093
35. Witoelar A, Jansen IE, Wang Y, et al (2017) Genome-wide pleiotropy between Parkinson disease and autoimmune diseases. JAMA Neurol 74:780–792. https://doi.org/10.1001/jamaneurol.2017.0469
36. Gelernter J, N. Sun, Polimanti R, et al (2019) Genome-wide Association Study of Posttraumatic Stress Disorder (PTSD) Re-Experiencing Symptoms in >165,000 US Veterans. Nat Neurosci 22:1394–1401. https://doi.org/10.1038/s41593-019-0447-7.Genome-wide
37. Khatami SG, Domingo-Fernandez D, Mubeen S, et al (2021) A Systems Biology Approach for Hypothesizing the Effect of Genetic Variants on Neuroimaging Features in Alzheimer's Disease. J Alzheimer's Dis 80:831–840. https://doi.org/10.3233/JAD-201397
38. Lin H, Zhu X, Chen G, et al (2017) KDM3A-mediated demethylation of histone H3 lysine 9 facilitates the chromatin binding of Neurog2 during neurogenesis. Dev 144:3674–3685. https://doi.org/10.1242/dev.144113
39. Zhang C, Qin F, Li X, et al (2022) Identification of novel proteins for lacunar stroke by integrating genome-wide association data and human brain proteomes. BMC Med 20:1–11. https://doi.org/10.1186/s12916-022-02408-y
40. Kourtian S, Soueid J, Makhoul NJ, et al (2017) Candidate Genes for Inherited Autism Susceptibility in the Lebanese Population. Sci Rep 7:1–9. https://doi.org/10.1038/srep45336

41. Choi MR, Han JS, Chai YG, et al (2020) Gene expression profiling in the hippocampus of adolescent rats after chronic alcohol administration. Basic Clin Pharmacol Toxicol 126:389–398. https://doi.org/10.1111/bcpt.13342
42. Huang YT, Zhang Y, Wu Z, Michaud DS (2017) Genotype-based gene signature of glioma risk. Neuro Oncol 19:940–950. https://doi.org/10.1093/neuonc/now288
43. Bharadwaj RA, Jaffe AE, Chen Q, et al (2018) Genetic risk mechanisms of posttraumatic stress disorder in the human brain. J Neurosci Res 96:21–30. https://doi.org/10.1002/jnr.23957
44. Al-Amri AH, Armstrong P, Amici M, et al (2022) PDZD8 Disruption Causes Cognitive Impairment in Humans, Mice, and Fruit Flies. Biol Psychiatry 92:323–334. https://doi.org/10.1016/j.biopsych.2021.12.017
45. Voigt D, Scheidt U, Derfuss T, et al (2017) Expression of the antioxidative enzyme peroxiredoxin 2 in multiple sclerosis lesions in relation to inflammation. Int J Mol Sci 18:. https://doi.org/10.3390/ijms18040760
46. Basso M, Giraudo S, Corpillo D, et al (2004) Proteome analysis of human substantia nigra in Parkinson's disease. Proteomics 4:3943–3952. https://doi.org/10.1002/pmic.200400848
47. Kato S, Kato M, Abe Y, et al (2005) Redox system expression in the motor neurons in amyotrophic lateral sclerosis (ALS): immunohistochemical studies on sporadic ALS, superoxide dismutase 1 (SOD1)-mutated familial ALS, and SOD1-mutated ALS animal models. Acta Neuropathol 110:101–112. https://doi.org/10.1007/s00401-005-1019-3
48. Yao J, Taylor M, Davey F, et al (2007) Interaction of amyloid binding alcohol dehydrogenase/Aβ mediates up-regulation of peroxiredoxin II in the brains of Alzheimer's disease patients and a transgenic Alzheimer's disease mouse model. Mol Cell Neurosci 35:377–382. https://doi.org/10.1016/j.mcn.2007.03.013
49. Palmfeldt J, Henningsen K, Eriksen SA, et al (2016) Protein biomarkers of susceptibility and resilience to stress in a rat model of depression. Mol Cell Neurosci 74:87–95. https://doi.org/10.1016/j.mcn.2016.04.001
50. Lexin W, Weiyi L, Ting T, et al (2022) Identification of novel biomarker and therapeutic target candidates for acute intracerebral hemorrhage by quantitative plasma proteomics. J Tradit Chinese Med 42:622–632. https://doi.org/10.1186/s12014-017-9149-x
51. Barber JCK, Rosenfeld JA, Foulds N, et al (2013) 8P23.1 Duplication Syndrome; Common, Confirmed, and Novel Features in Six Further Patients. Am J Med Genet Part A 161:487–500. https://doi.org/10.1002/ajmg.a.35767
52. Zhang M, Ding X, Zhang Q, et al (2021) Exome sequencing of 112 trios identifies recessive genetic variants in brain arteriovenous malformations. J Neurointerv Surg 13:568–573. https://doi.org/10.1136/neurintsurg-2020-016469
53. Pettegrew JW, Levine J, McClure RJ (2000) Acetyl-L-carnitine physical-chemical, metabolic, and therapeutic properties: Relevance for its mode of action in Alzheimer's disease and geriatric depression. Mol Psychiatry 5:616–632. https://doi.org/10.1038/sj.mp.4000805
54. Häggmark A, Mikus M, Mohsenchian A, et al (2014) Plasma profiling reveals three proteins associated to amyotrophic lateral sclerosis. Ann Clin Transl Neurol 1:544–553. https://doi.org/10.1002/acn3.83
55. Hemby SE, Ginsberg SD, Brunk B, et al (2002) Gene Expression Profile for Schizophrenia. Arch Gen Psychiatry 59:631. https://doi.org/10.1001/archpsyc.59.7.631
56. Kunii M, Noguchi Y, Yoshimura SI, et al (2021) SNAP23 deficiency causes severe brain dysplasia through the loss of radial glial cell polarity. J Cell Biol 220:. https://doi.org/10.1083/JCB.201910080
57. Di C, Liang J, Wang Y, et al (2021) SPZ1 promotes glioma aggravation via targeting CXXC4. J BUON 26:373–379

58. Cruceanu C, Kutsarova E, Chen ES, et al (2016) DNA hypomethylation of Synapsin II CpG islands associates with increased gene expression in bipolar disorder and major depression. BMC Psychiatry 16:1–5. https://doi.org/10.1186/s12888-016-0989-0
59. Larson ME, Greimel SJ, Amar F, et al (2017) Selective lowering of synapsins induced by oligomeric α-synuclein exacerbates memory deficits. Proc Natl Acad Sci U S A 114:E4648–E4657. https://doi.org/10.1073/pnas.1704698114
60. Kipkorir T, Colangelo CM, Manuelidis L (2015) Proteomic analysis of host brain components that bind to infectious particles in Creutzfeldt-Jakob disease. Proteomics 15:2983–2998. https://doi.org/10.1002/pmic.201500059.Proteomic
61. Grebb JA, Greengard P (1990) An Analysis of Synapsin II, a Neuronal Phosphoprotein, in Postmortem Brain Tissue from Alcoholic and Neuropsychiatrically Ill Adults and Medically Ill Children and Young Adults. Arch Gen Psychiatry 47:1149–1156. https://doi.org/10.1001/archpsyc.1990.01810240069011
62. Delprato A, Bonheur B, Algéo MP, et al (2015) Systems genetic analysis of hippocampal neuroanatomy and spatial learning in mice. Genes, Brain Behav 14:591–606. https://doi.org/10.1111/gbb.12259
63. Domanico D, Fragiotta S, Cutini A, et al (2015) Psychosis, Mood and Behavioral Disorders in Usher Syndrome: Review of the Literature. Med hypothesis, Discov Innov Ophthalmol J 4:50–5
64. Filatova E V., Shadrina MI, Alieva AK, et al (2014) Expression analysis of genes of ubiquitin-proteasome protein degradation system in MPTP-induced mice models of early stages of Parkinson’s disease. Dokl Biochem Biophys 456:116–118. https://doi.org/10.1134/S1607672914030107
65. Peppercorn K, Kleffmann T, Jones O, et al (2022) Secreted Amyloid Precursor Protein Alpha, a Neuroprotective Protein in the Brain Has Widespread Effects on the Transcriptome and Proteome of Human Inducible Pluripotent Stem Cell-Derived Glutamatergic Neurons Related to Memory Mechanisms. Front Neurosci 16:1–23. https://doi.org/10.3389/fnins.2022.858524
66. Baumgart M, Groth M, Priebe S, et al (2014) RNA-seq of the aging brain in the short-lived fish N. furzeri - conserved pathways and novel genes associated with neurogenesis. Aging Cell 13:965–974. https://doi.org/10.1111/acel.12257
67. Naef V, Monticelli S, Corsinovi D, et al (2018) The age-regulated zinc finger factor ZNF367 is a new modulator of neuroblast proliferation during embryonic neurogenesis. Sci Rep 8:1–11. https://doi.org/10.1038/s41598-018-30302-2
